# Navigation through the Plasma Membrane Molecular Landscape Shapes Random Organelle Movement

**DOI:** 10.1016/j.cub.2016.12.002

**Published:** 2017-02-06

**Authors:** Alison R. Dun, Gabriel J. Lord, Rhodri S. Wilson, Deirdre M. Kavanagh, Katarzyna I. Cialowicz, Shuzo Sugita, Seungmee Park, Lei Yang, Annya M. Smyth, Andreas Papadopulos, Colin Rickman, Rory R. Duncan

**Affiliations:** 1 Institute of Biological Chemistry, Biophysics and Bioengineering, Heriot-Watt University, Edinburgh EH14 4AS, UK; 2 Edinburgh Super-Resolution Imaging Consortium; 3 Department of Mathematics, Maxwell Institute, MACS, Heriot-Watt University, Edinburgh EH14 4AS, UK; 4 Toronto Western Research Institute, Room 11-432, McLaughlin Wing, 399 Bathurst St., Toronto, ON M5T 2S8, Canada; 5 Centre for Inflammation Research, University of Edinburgh, The Queen’s Medical Research Institute, 47 Little France Crescent, Edinburgh EH16 4TJ, UK; 6 The Clem Jones Centre for Ageing Dementia Research, Queensland Brain Institute, The University of Queensland, Brisbane, QLD 4072, Australia

**Keywords:** vesicle, exocytosis, super-resolution, microscopy, mathematical modeling, actin, cytoskeleton

## Abstract

Eukaryotic plasma membrane organization theory has long been controversial, in part due to a dearth of suitably high-resolution techniques to probe molecular architecture in situ and integrate information from diverse data streams [1]. Notably, clustered patterning of membrane proteins is a commonly conserved feature across diverse protein families (reviewed in [2]), including the SNAREs [3], SM proteins [4, 5], ion channels [6, 7], and receptors (e.g., [8]). Much effort has gone into analyzing the behavior of secretory organelles [9, 10, 11, 12, 13], and understanding the relationship between the membrane and proximal organelles [4, 5, 12, 14] is an essential goal for cell biology as broad concepts or rules may be established. Here we explore the generally accepted model that vesicles at the plasmalemma are guided by cytoskeletal tracks to specific sites on the membrane that have clustered molecular machinery for secretion [15], organized in part by the local lipid composition [16]. To increase our understanding of these fundamental processes, we integrated nanoscopy and spectroscopy of the secretory machinery with organelle tracking data in a mathematical model, iterating with knockdown cell models. We find that repeated routes followed by successive vesicles, the re-use of similar fusion sites, and the apparently distinct vesicle “pools” are all fashioned by the Brownian behavior of organelles overlaid on navigation between non-reactive secretory protein molecular depots patterned at the plasma membrane.

## Highlights

• Membrane-proximal vesicle movements are random
• Secretory molecules at the plasma membrane organize into conformational clusters
• Random vesicle movements navigate among these clusters
• This model explains vesicle behaviors without a requirement for functional pools

## Graphical Abstract

**Figure:**
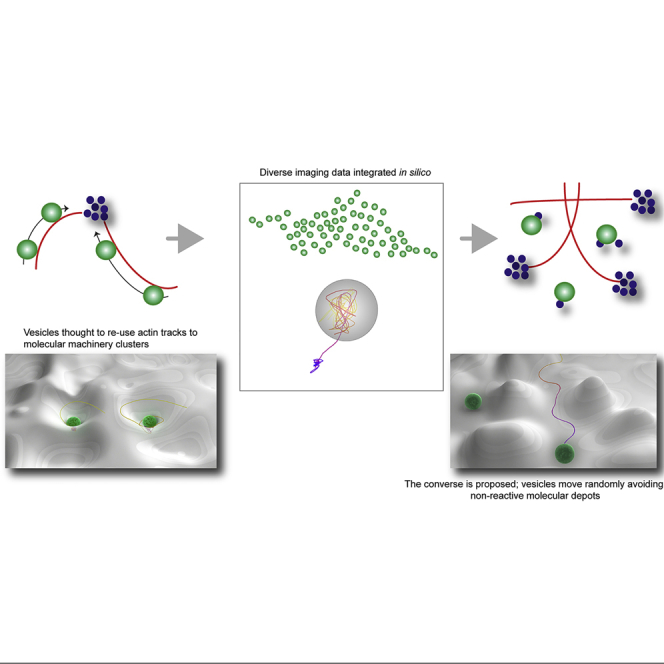

## Teaser

Dun et al. use mathematical modeling, super-resolution imaging, and biological perturbations to shape the membrane molecular landscape, examining the inter-relationship between the plasma membrane and organelle dynamics. They show that secretory vesicles at the cell surface move randomly, navigating among depots of secretory machinery molecules.

## Results and Discussion

**Figure 1:**
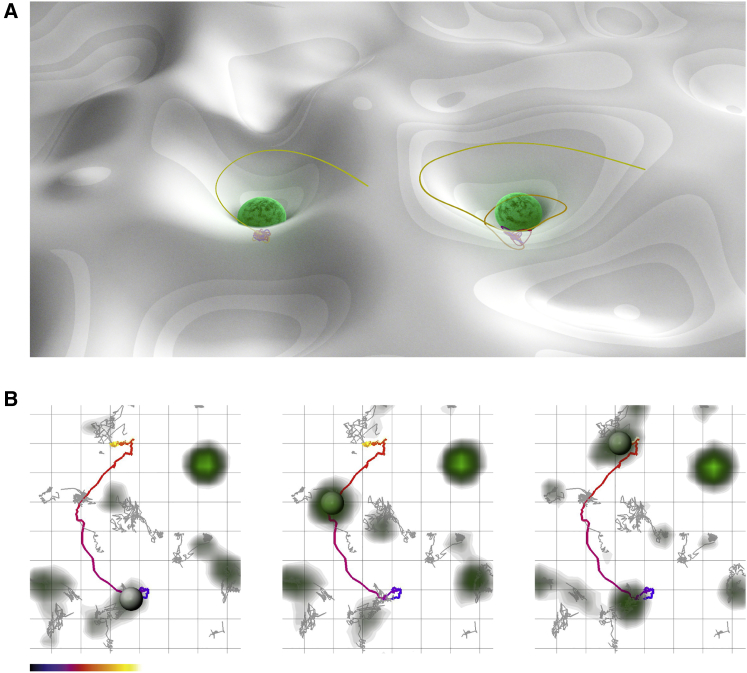
Current Model for Vesicle Dynamics at the Membrane (A) Stylized cartoon showing a model for vesicle dynamics at the plasma membrane, where LDCVs are docked on molecular machinery depots. This is illustrated as a “well,” attracting LDCVs. (B) Images from a TIRFM recording of a PC12 cell expressing lumenal NPY-EGFP. A single vesicle (gray sphere) is shown (track in color) scanning the membrane, visiting areas preferred by other vesicles (gray tracks). Grid scale, 500 nm grid edge. Color bar shows time. See also Figure S1 and Movie S1.

To address the question of whether membrane-proximal vesicles behave in a controlled manner, we first posed a simple question: when new vesicles are recruited to the plasma membrane, is this spatially random, or is there some order to this process? We labeled large dense-core vesicles (LDCVs) in secretory cells (phaeochromocytoma cells, PC12s) using soluble cargo Neuropeptide Y (NPY) fused to EGFP [17, 18]. We stimulated cells to secrete and then followed the recruitment of new LDCVs using total internal reflection fluorescence microscopy (TIRFM). We quantified trajectories taken by all vesicles before, during, and after exocytosis. This marked the image plane of the plasma membrane with areas visited by LDCVs during the recording period and allowed us to determine the arrival sites of any new recruits. Figure 1B displays frames from Movie S1, showing a vesicle arriving on top of a site occupied by an earlier LDCV, scanning the same region before moving off to visit at least two other previously occupied regions. The area under scrutiny is ∼4 μm^2^. We found LDCVs follow similar trajectories to similar (but not identical) fusion sites on the cell surface (Figures S2A–S2D), even after treatment with Methyl-β-cyclodextrin to quantifiably disrupt plasma membrane lipid order [19] (Figures S2E–S2G). These data appear to support current models, suggesting that LDCVs visit preferred sites on the membrane, using defined, re-usable routes that intuitively appear like physical tracks.

We previously used photoactivated localization microscopy (PALM) [20, 21] and direct stochastic optical reconstruction microscopy (dSTORM) [22] to relate syntaxin1a and SNAP-25 molecular positions with those of single LDCVs [4, 5, 18]. We examined mCherry molecule aggregation in living cells, to exclude the possibility that the non-uniform patterning of the SNARE fusions at the plasma membrane could be caused by fluorescent protein oligomerization. For this we used fluorescence correlation spectroscopy, as we have before [4], in primary cells and cell lines, finding no evidence of aggregation of fusions or unfused mCherry.

We found previously that the number of tSNARE/SM molecules residing within functionally relevant distances of LDCVs near the plasma membrane is very low, with LDCVs encountering small numbers of isolated tSNAREs/SM proteins located between molecular clusters or depots. This was confirmed using stimulated emission deletion (microscopy; STED) imaging of endogenous SNAREs, finding immuno-labeled LDCVs surrounded by tSNARE clusters [17]. Similar results were found for the SM protein and syntaxin1a chaperone munc18-1 [5], marrying imaging with biophysics techniques that showed small numbers of SNARE/SM molecules are required to drive vesicle fusion.

We created density maps of the cell surface from vesicle dynamics data, finding that the pattern of vesicle trajectories was non-uniform over the cell surface, with many sites connected by common tracks, visited by multiple vesicles. This presented a conundrum: if LDCVs move along re-usable paths, revisiting specific membrane sites, what do these apparent routes connect, if not SNARE/SM depots?

**Figure 2:**
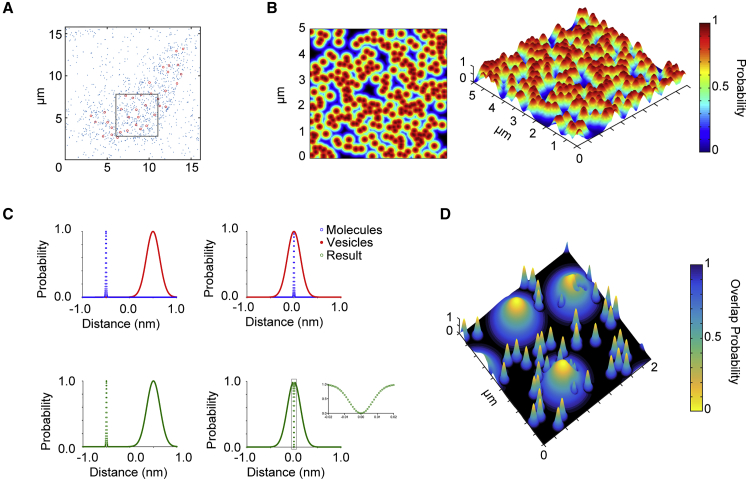
Probability of Syntaxin1a Molecule/LDCV Functional Overlap Is Low (A) Molecule and vesicle positions derived from real data, with molecules represented in blue and vesicles in red. (B) Molecular densities plotted for a 5 μm^2^ region of plasma membrane, illustrating the probability of molecules being present, represented in 2D (left) and 3D (right) plots. (C) A method to measure overlap is shown graphically, using synthetic data, with molecules in red, vesicles in blue, and the result of combining the two in green, where the probability of a molecule localization is subtracted from the probability of a vesicle position (described in the Supplemental Experimental Procedures). (D) Vesicles are predominantly in molecular gaps and overlap probability with SNARE/SM depots is low. See also Figures S2 and S4.

To address this, we modeled data in silico, integrating quantitative information describing molecular numbers, positions, and densities with dynamic datasets describing similar parameters for LDCVs, iterating with knockdown cells to perturb the biological system and compare with predictions made by our mathematical model. We considered vesicles in a potential field formed by tSNARE and SM proteins. Munc18-1 is present at the membrane only by virtue of a 1:1 stoichiometry interaction with the tSNARE, syntaxin1, or the tSNARE heterodimer [4], so the potential fields were constructed from peaks of a two-dimensional Gaussian function centered on each molecular coordinate describing munc18-1 location. This was informed using PALM data from munc18-1-null cells, rescued functionally by PA-mCherry-munc18-1 [5] (Figures 2A and 2B). The model is deliberately simple, but it displays key qualitative and quantitative features that indicate it might be a good match to the biological data. Our aim was not a precisely calibrated model, but rather to show that this standard incomplete model can explain the observed behavior of vesicles in living cells as well as predict biological outcomes following perturbations.

Vesicle positions determined from corresponding TIRFM data were added, incorporating information about vesicle dynamics from those experiments; i.e., arrival position, movement, speed, etc. We further informed our model with a probability field for the positions of the vesicles with an in-plane radius of 82.5 nm, as we explained before [18]. This represents a distance from the inner leaflet of the plasma membrane where membrane-proximal LDCVs can interact with tSNARE molecules. We then considered the probability of functional overlap between the molecules and the vesicles, modeling real data and examining the probability that vesicles and molecules meet (Figures 2C and 2D). This supported the imaging experiments, indicating a very low probability of LDCV and tSNARE/SM molecule interaction; importantly, at diffraction-limited resolution, “colocalization” is common, but this cannot be functionally relevant [18].

We questioned whether LDCVs move along actin filaments, as sometimes suggested. To do this, we acquired dual-color TIRFM images from living cells expressing Lifeact [23]-EGFP and NPY-mCherry. These experiments showed no correlation between actin features and vesicle trajectories (Figures S3A–S3D). We perturbed the actin cytoskeleton using Latrunculin A, resulting in partial rearrangements in Lifeact-labeled structures. We found no effect of this intervention on the vesicle behaviors we measured (Figures S3E–S3H). To investigate this further, we used confocal laser-scanning microscopy (CLSM), visualizing the actin cortical layer, LDCVs, and the plasma membrane in the same samples. Combined with image data deconvolution, this provides lateral resolution of around 200 nm, sufficient to resolve potentially separate arrangements of these structures. Equatorial sections in these images confirmed that LDCVs near the cell surface appear embedded within the cortical layer (as reviewed in [24]), but, importantly, vesicles there reside in “spaces” in the actin network (Figure S3I). This is impossible to assess in CLSM equatorial sections, so we next performed TIRFM on the same samples. This analysis revealed that, indeed, membrane-proximal LDCVs reside in windows in the cortical actin network that are larger than the longest walks taken by membrane-proximal vesicles. Together, these experiments show directly that LDCVs at the membrane do not follow actin fibers, nor are they constrained necessarily by the cortical network there (Figures S3J–S3L).

Thus, we re-visited the current understanding of vesicle dynamics. We attempted to replicate the use of repeated routes connecting tSNARE/SM depots by LDCVs (Figure 1; Movies S1, S2, and S3) in silico by creating “potential wells” defining tSNARE/SM protein positions and densities, attracting modeled vesicles. Biologically, this could be explained by vesicles being directed to sites of molecular interaction, representing most current understanding.

In agreement, this rehearsal of the mathematical model did not reiterate the biology (nor did it represent the molecule/vesicle probability overlap revealed by nanoscopy). Instead, we turned this problem around and asked what would happen if the protein depots were *avoided* by the model vesicles.

**Figure 3:**
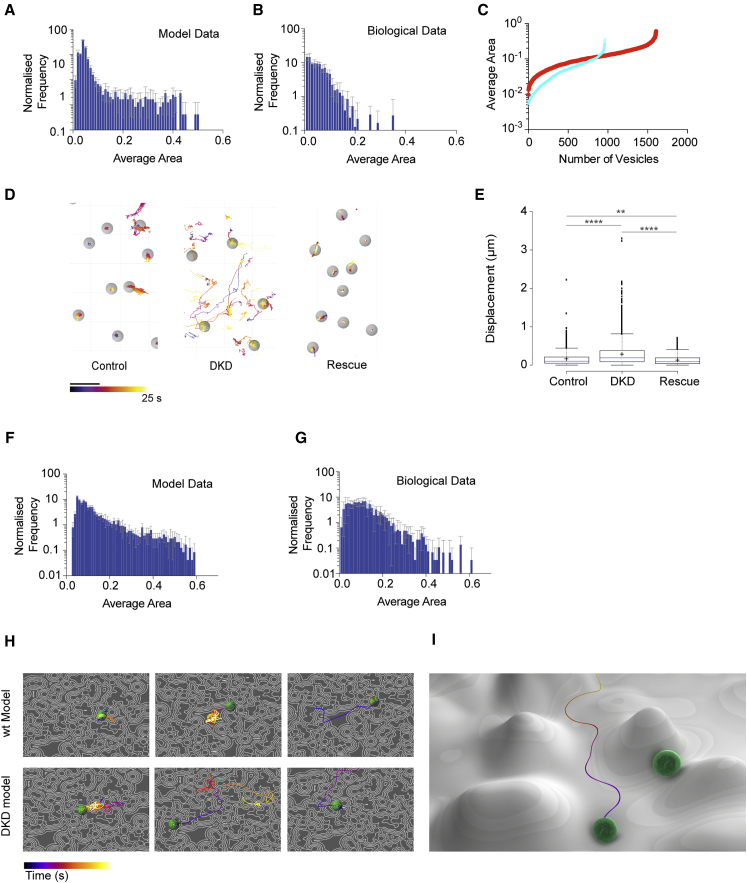
Modeling LDCV Dynamics In Silico Reiterates Biological Data Only if Vesicles Avoid Secretory Machinery Depots (A) The average area sampled by vesicles, generated from 2,400 in silico vesicles over four iterations. (B) A similar measurement for real biological data (941 vesicles in four cells) reiterates the mathematical model. Error bars show the SD. (C) The distribution of real vesicle dynamics follows a continuum with no statistically distinct sub-pools of vesicle behavior (wild-type [WT], blue; mutant [DKD], red). (D) Images show vesicle tracks within an area of plasma membrane. Left, WT cells; middle, DKD M18 cells; right, DKD M18 cells rescued with munc18-1. Gray spheres (vesicles) are 400 nm diameter. Scale bar, 1 μm. Color scale for tracks spans 25 s. (E) Boxplots show displacement for all vesicles tracked in WT PC12, DKD M18, and control DKD M18 cells rescued with munc18-1. Line shows median displacement, with outliers presented. Kruskal-Wallis test, ^∗∗∗∗^p > 0.0001 and ^∗∗^p > 0.01. (F) The average area sampled by in silico vesicles, generated from our mutant model for 2,400 vesicles from four iterations. (G) The same average area measurement for real biological data from DKD cells (1,606 vesicles from four cells). Error bars show the SD. (H) In silico vesicles show a variation in dynamics (suggestive of “immobile” or “scanning” as previously suggested [25]), but with all behaviors falling on the same continuum shown in (C). Top panels show in silico vesicles from the WT model (see also Movie S3), and bottom panels show results when molecular depots are depleted (to mimic DKD cells). (I) Stylized cartoon showing our model for vesicle dynamics at the plasma membrane, where LDCVs avoid molecular machinery depots. This is illustrated as vesicles following valleys. See also Figure S3.

We computed the dynamic behavior of 600 virtual vesicles in four replicates, starting with the initial positions and numbers of vesicles and molecules observed in the real biological data. In this scenario, the in silico vesicle behavior reiterated the real biology rather well, with vesicles navigating along “valleys” between the molecular densities, buffeted in an otherwise Brownian manner (Figures 3A and 3B). This finding was encouraging, providing an alternative description for dynamics seen at the cell surface; no physical tracks need be present to explain LDCV saltatory movements, changing speeds, the apparent re-use of trajectories, or re-sampling of membrane sites. Using this theory, all vesicle behaviors can be described with a single continuum (Figure 3C).

We expected that if exocytotic protein machinery depots were depleted from the cell surface, so the molecular “valleys” that shape the LDCV trajectories would also be altered; in this case, LDCV movements should shift toward Brownian behavior. Disrupting munc18-1 expression, while invaluable [26], has been problematic for two reasons; first, munc18-1 and syntaxin1 expression levels, functions, and localizations are intimately linked, meaning that disrupting one affects the other [27]. Second, munc18-2 can compensate for munc18-1 in cells and in null animals [28]. We therefore used munc18-1/2 double knockdown (DKD) cells shown to lack munc18 function [28]. Munc18-1, syntaxin1a, and syntaxin1a/SNAP-25 heterodimer are all postulated to be “docking factors” in the literature [26, 29, 30], illustrating the inter-linked nature of their biology. Having suggested that LDCVs navigate between tSNARE/SM depots, we wanted to determine whether this was due to the presence of syntaxin1 or Munc18 molecules or whether both are required. To look at this, we performed dSTORM, immuno-localizing syntaxin1 molecules that reached the cell surface in munc18-1/2 DKD cells. This revealed that plasma membrane syntaxin1 molecules are present with a similar distribution to that in wild-type cells (Figure S4). Performing vesicle-tracking experiments agreed with our prediction; membrane-proximal vesicles in these cells adopted an increased Brownian motion compared to control cells or to DKD cells rescued with heterologous munc18-1 expression, with longer walks, using unique paths (Figures 3D and 3E). We modified our model parameterization to reduce the magnitude of molecular depots accordingly and found that in silico vesicle dynamics reiterated the real biology (Figures 3G–3I). This echoed the idea that whereas our model is not precisely quantitative, it can predict the effects of specific molecular perturbations on the dynamics of intracellular organelles (Table S1). These experiments suggested that if the equilibrium of reactive versus non-reactive SNAREs is altered (e.g., by depletion of SM proteins), then LDCV behavior is altered in a predictable way (Figure 3J).

**Figure 4:**
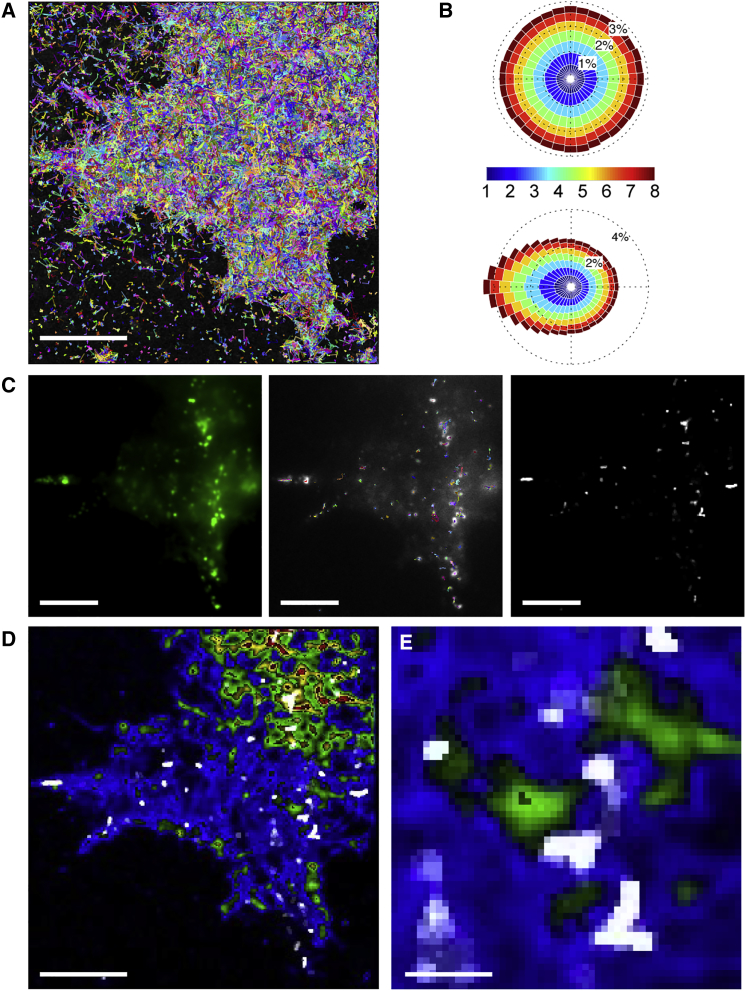
LDCVs in Living Cells Navigate among tSNARE Molecular Depots Containing munc18 Proteins (A) Single-particle trajectories of 245,964 syntaxin1a molecules in a living cell. Track color is only for contrast. (B) Rose diagrams are angular histograms with 36 bins, each of 10°. Histogram bin magnitude indicates the number of molecule tracks with that direction relative to normal, and color is molecular speed. This shows that tSNARE molecules behave in a Brownian manner with complete freedom of direction in their initial movements, illustrated as a symmetrical (circular) histogram (top). If a preceding direction is known from tracking data, the next step in molecular direction is frequently a reversal, shown by a skewing to the left in the Rose diagram (bottom). (C) LDCVs labeled with lumenal NPY-EGFP in the same cell (left). Vesicles were tracked (middle) with track color for contrast. Trajectory information was converted into a density map, representing the number of LDCV tracks crossing each pixel in the image over time (right; white densities). (D and E) The LDCV track density (D, white) was overlaid on a density map (color) of syntaxin1a molecule tracks, with the boxed area magnified in (E). These experiments show directly that LDCVs navigate paths among tSNARE depots. Scale bars, 5 μm (A, C, and D) and 1 μm (E). See also Figure S4.

Finally, we returned to live-cell imaging to examine directly the vesicle dynamics in relation to tSNARE/SM depots. We acquired data from NPY-EGFP-labeled LDCVs, followed immediately by single-particle tracking-PALM (sptPALM [31]). We used our sptPALM algorithms [4, 5, 18, 32] to provide high-density trajectory maps, super-imposing the LDCV-tracking data onto these. A sample of these data, from four independent experiments, is shown in Figure 4A, displaying 245,964 syntaxin1a-PAmCherry molecular tracks in a single cell. We analyzed the vectors taken by every tSNARE in the dataset, finding that molecules have complete freedom of movement in their initial movements (Figure 4B, top; see also Figure S1), consistent with Brownian diffusion in the membrane plane. We next examined the course followed once a molecule is already moving, finding that the tSNARE molecules behave as if caged in the membrane, with frequent reversals in direction (Figure 4B, bottom; as we previously showed using different techniques and in different cell types in multiple [n > 15] experiments [4, 5, 18]).

Next, we examined LDCV position in the same samples (Figure 4C, left), tracking each single LDCV as before (Figure 4C, middle). We converted tracks into density maps, showing the frequency that an LDCV crossed each pixel in the image (Figure 4C, right). We super-imposed this onto a map describing the density of all tSNARE molecular tracks (Figure 4D), demonstrating that LDCV tracks are contained in the valleys between tSNARE depots, confirming our hypothesis and validating our model.

The combination of data from different imaging modalities remains challenging, as does the interpretation of the data in the biological context beyond simply locational information. Here, we used single-molecule localization coordinate data to inform a mathematical model, integrating information from a variety of imaging and spectroscopic approaches in silico, where such analysis is not possible in the real world.

We propose that clustering of the tSNARE proteins, alongside spatially modulated SM protein sequestration of syntaxin molecules at the membrane [14], creates landscapes of non-reactive plasma membrane [33] interspersed with reactive SNAREs that LDCVs navigate. We have shown before that the tSNARE heterodimer is largely assembled at the plasma membrane [14], that munc18-1 is present at the plasma membrane by virtue of interaction with either monomeric syntaxin1, or the heterodimer [34], and that this interaction is maintained throughout exocytosis [4]. We [17] and others [35] have shown that the composition of the SNARE/SM cluster may vary with spatial location at the cell surface and that these clusters are interspaced with smaller numbers of SNARE and SM proteins [5]. The patterning of SNARE proteins and vesicle recruitment has been shown to be ordered by phosphatidylinositol 4,5-bisphosphate (PIP2) [36]; further work may reveal the non-protein membrane components, such as PIP2, control vesicle dynamics and movements, as well as “docking.” We show little spatial correlation between actin networks and LDCV trajectory; nevertheless, the role of the actin cortical layer in regulating the supply of vesicles to the membrane is clear [12]. Recent work suggested that stable SNARE depots themselves are anchored by the actin cytoskeleton [37]. Our work complements these studies, and we propose that the underlying membrane composition, combined with actin and clustering of membrane proteins, shapes the Brownian motion of membrane-proximal organelles. This theory apparently contrasts with some earlier works [10], probably because of the higher spatial resolutions accessed here that show that “colocalization” cannot correlate with function [5, 18]. Here, we have not looked at fusion sites with molecular resolution, and so it may be that at the site of exocytosis, rapid rearrangements of the secretory machinery occur, as earlier studies suggest [10, 38]. The behavior of secretory vesicles and other organelles at the plasma membrane has never been explained adequately, with current models relying on subjective categorization of apparent behaviors into sub-groups. This in turn correlates with models describing different stages of the secretory pathway that are also not entirely satisfactory. The non-uniform spatial and functional patterning of tSNARE molecules at the plasma membrane, organized by the underlying lipid composition, has been shown to be essential for neurotransmission by guiding synaptic vesicles to active membrane areas [16]. Here we show the effect that this has on LDCV dynamics, providing a unifying model that can describe all apparent vesicle behaviors.

## Experimental Procedures

The materials and methods are given in the Supplemental Experimental Procedures.

## Author Contributions

A.R.D., A.M.S., C.R., R.S.W., A.P., D.M.K., K.I.C., L.Y., and R.R.D. acquired and analyzed the data. G.J.L. proposed the mechanism of the model, examined the overlap, and performed the simulations. S.S. and S.P. generated the DKD cells. R.R.D., S.S., A.R.D., and C.R. designed the experiments. R.R.D. and A.R.D. wrote the manuscript.

## Supplemental Information

Document S1. Supplemental Experimental Procedures, Figures S1–S4, and Table S1

Movie S1. A Mobile Vesicle, Related to Figure 1B A mobile vesicle (gray sphere and colored track) scanning the membrane over sites previously visited by other vesicles (gray tracks). Grid scale: 500 nm grid edge, color scale shows 0–28 s, frame rate: 40 Hz.

Movie S2. Newly Recruited Vesicles Arriving at the Plasma Membrane, Related to Figure 3 Newly recruited vesicles arriving at the plasma membrane (gray sphere, colored track) and moving along similar paths to similar fusion sites. Previous vesicle tracks (gray), grid scale: 500 nm grid edge, color scale shows 0–41 s, frame rate: 40 Hz.

Movie S3. A Typical In Silico Vesicle, Related to Figure 3 The large green circle is 400 nm in diameter and represents the LDCV diameter. The smaller circle inside this is 165 nm diameter and represents the meaningful “functional” diameter of an LDCV that can sample the membrane molecular landscape (described in [S4]). Gray and white contours represent molecular mountains as shown in Figure 3, color scale shows 0–24 s, frame rate: 40 Hz.

Document S2. Article plus Supplemental Information
