# Supplementary material for: Navigation through the Plasma Membrane Molecular Landscape Shapes Random Organelle Movement
Source: Curr Biol. 2017 Feb 6;27(3):408–14. doi: 10.1016/j.cub.2016.12.002 (PMC5300901; doi:10.1016/j.cub.2016.12.002)
Supplement: Document S1. Supplemental Experimental Procedures, Figures S1–S4, and Table S1 [file mmc1.pdf]

Current Biology, Volume *27*

Supplemental Information

Navigation through the Plasma Membrane Molecular Landscape Shapes Random Organelle Movement

Alison R. Dun, Gabriel J. Lord, Rhodri S. Wilson, Deirdre M. Kavanagh, Katarzyna I. Cialowicz, Shuzo Sugita, Seungmee Park, Lei Yang, Annya M. Smyth, Andreas Papadopulos, Colin Rickman, and Rory R. Duncan

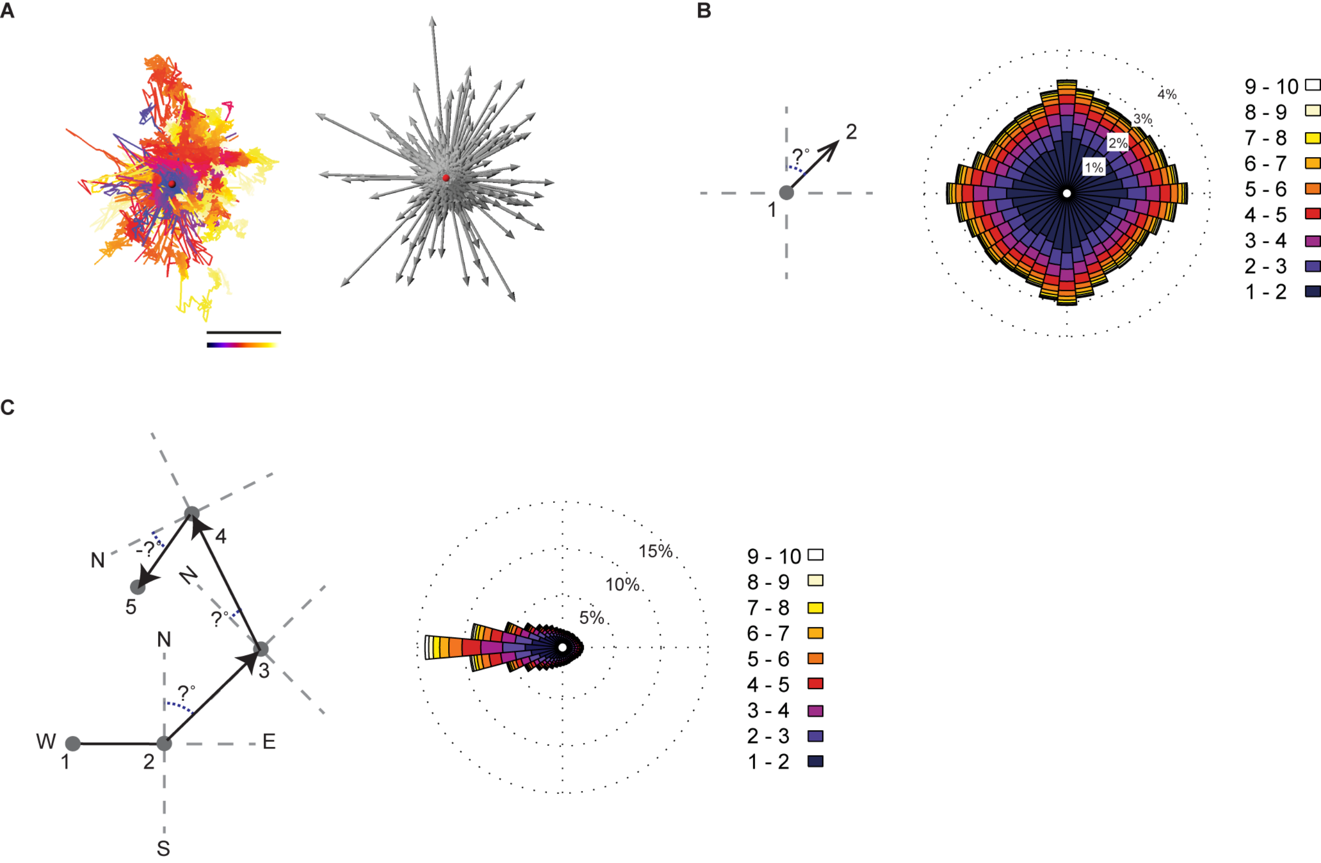
A
B
1
2
?°
4%
3%
2%
1%
9 - 10
8 - 9
7 - 8
6 - 7
5 - 6
4 - 5
3 - 4
2 - 3
1 - 2
C
N
-?°
4
5
N
?°
N
3
?°
W
E
1
2
S
15%
10%
5%
9 - 10
8 - 9
7 - 8
6 - 7
5 - 6
4 - 5
3 - 4
2 - 3
1 - 2

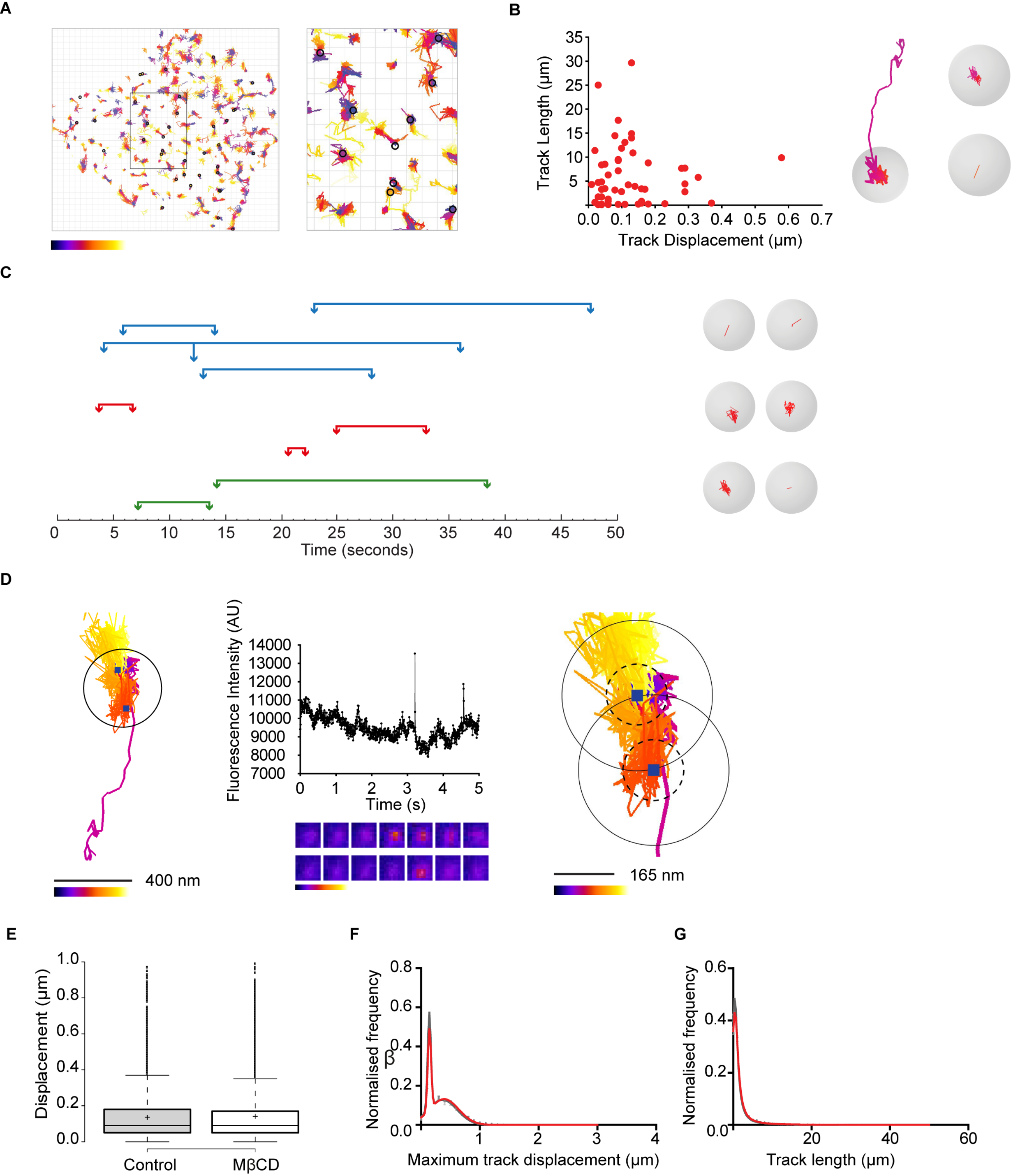

A
B
Track Length (μm)
Track Displacement (μm)
C
Time (seconds)
D
Fluorescence Intensity (AU)
Time (s)
400 nm
165 nm
E
Displacement (μm)
Control
MβCD
F
Normalised frequency
Maximum track displacement (μm)
G
Normalised frequency
Track length (μm)

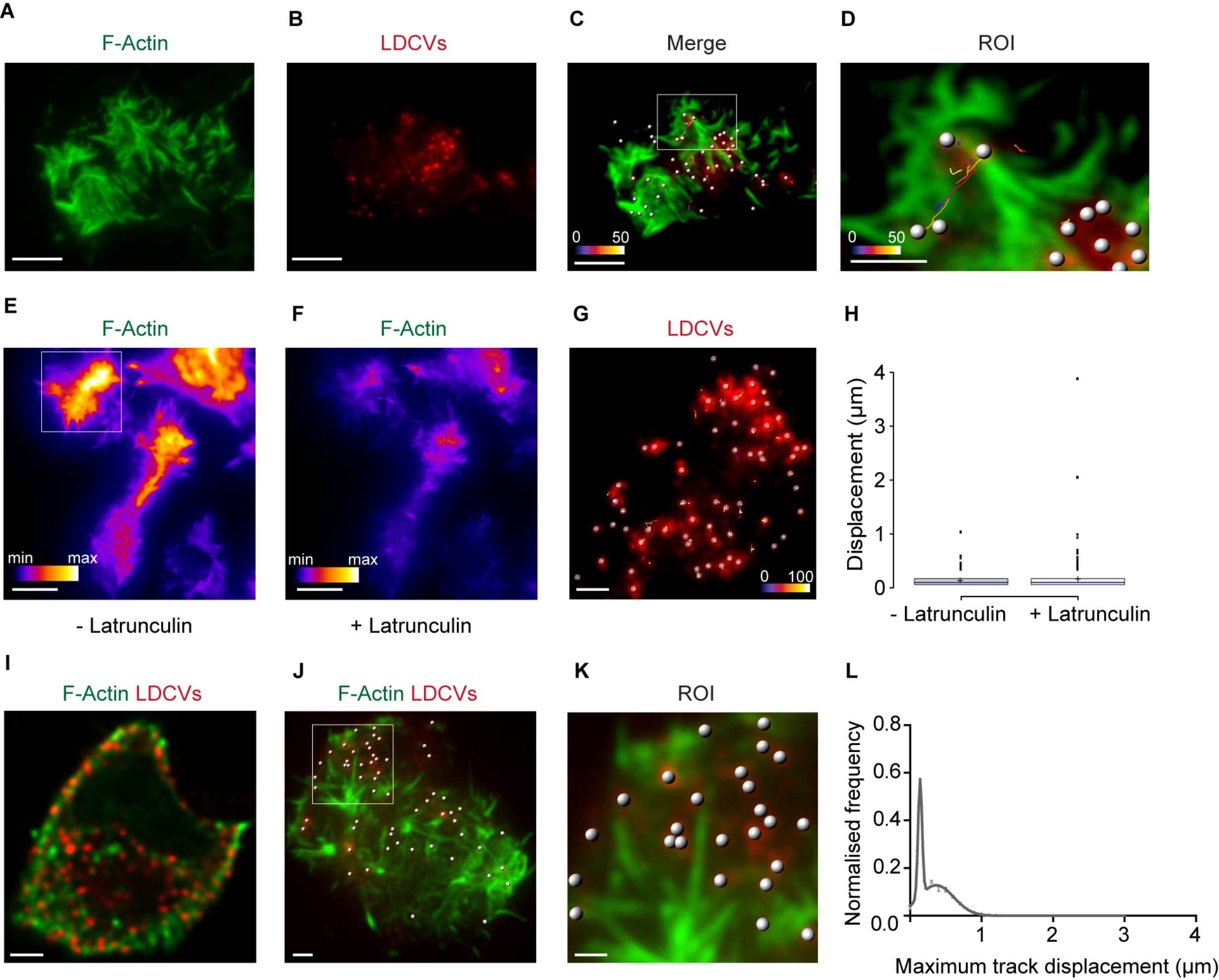

A
F-Actin
B
LDCVs
C
Merge
0
50
D
ROI
0
50
E
F-Actin
min
max
- Latrunculin
F
F-Actin
min
max
+ Latrunculin
G
LDCVs
0
100
H
Displacement (μm)
4
3
2
1
0
- Latrunculin
+ Latrunculin
I
F-Actin LDCVs
J
F-Actin LDCVs
K
ROI
L
Normalised frequency
0.8
0.6
0.4
0.2
0.0
1
2
3
4
Maximum track displacement (μm)

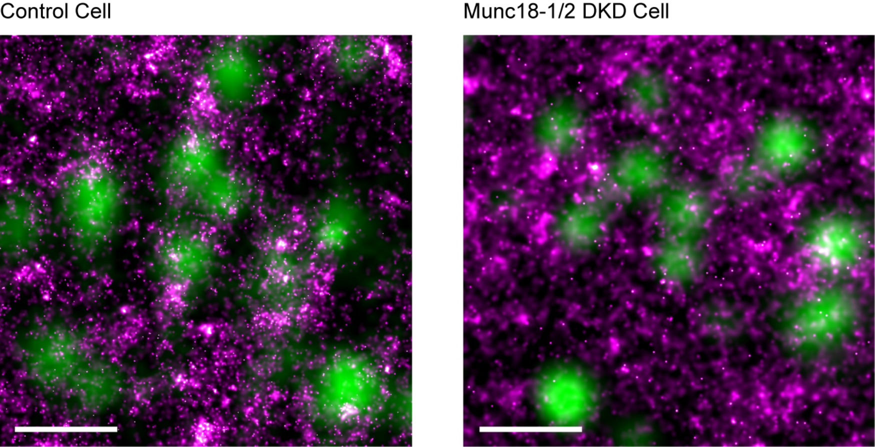
Control Cell
Munc18-1/2 DKD Cell

Supplemental Figure Legends

Figure S1 Vesicle mobility can be described by a Brownian model (related to Figure 1). A. Vesicle tracks (829) from a representative PC12 cell over 100 seconds, translated to start at the same xy coordinate. Coloured vesicle tracks (left) and track displacement displayed as arrows (right). Scale bar 1 µm. B. the angle and speed of the first movement taken by a vesicle is extracted (left; vesicle; grey circle, movement; black arrow, angle; blue dashed line) and displayed as a histogram (right) where colour represents speed (µm s^{-1}) and length of the bin represents frequency (as described in figure 4). Histogram shows 16,944 tracks from 5 representative PC12 cells. C. The angle and speed of the subsequent movements a vesicle makes in relation to its previous is extracted (left; as in b with subsequent steps numbered and compass points used for perspective) and displayed as a histogram (as in b). Colours indicate binned speeds, units are microns per second.

Figure S2 Vesicles fuse at frequently visited sites on the plasma membrane (related to Figure 2). Where appropriate, cells were stimulated to secrete with 300 µM ATP, added to the bath after 500 frames imaging at 20 Hz, with imaging continued for 1000 further frames. Secretion was visualised using TIRFM, by observing 'flashes' as luminal, acidic NPY-EGFP is exposed to the higher, extracellular pH. A. tracked vesicles (coloured tracks) at the membrane of a representative PC12 cell are shown, black circles show vesicle fusion sites within a 45 second recording, grid shows scale 400 nm. Black box indicates zoomed region (right panel). B. Track length against track displacement for vesicles that fused shows range of behaviour, example tracks shown (grey spheres show vesicle). C. arrow heads represent points of fusion along the scale with cell stimulation at 0 seconds, line shows refractory period until subsequent fusion in same 400 nm^2 region. Example tracks (red) within vesicles (grey sphere) show these vesicle pairs can immediately fuse (blue, top right), show caged behaviour (red, middle right) or a combination (green, lower right). D. (left panel) two vesicle fusion sites within 400 nm radius (solid circle), (middle panel) profile of fluorescence intensity over time (colour represents intensity). (right panel) fusion sites using radius of 82.5 nm (dashed line) are separate. All vesicle tracks are colour-coded to show time from 0 – 45 seconds (excluding C). E. Quantification of the effect of MβCD on PC12 cells. Box plots show displacement for all vesicles tracked inside wt PC12 and MβCD-treated PC12 cells (5 control cells and 6 MβCD-treated PC12 cells). Line shows median displacement, cross hair shows mean displacement and outliers are presented. F - G. maximum track displacement and track length shown from PC12 cells (grey) and MβCD-treated PC12 cells (red). Data normalised to number of tracks per cell (mean +/- SEM) n.s between control and treated in either parameter using a Mann-Whitney test.

Figure S3 Actin and vesicle imaging at the plasma membrane (related to Figure 3). A. F-actin and B. LDCVs at the plasma membrane of wtPC12 cells under TIRF illumination. C. a merge of both components with vesicles tracked at 20 Hz. Scale bar 5 µm. D. zoom of region of interest showing vesicles moving perpendicular to actin fibres, scale bar 2 µm. Tracks coloured as per colour bar from 0 to 50 frames. E. F-actin in wtPC12 cells under TIRF illumination before Latrunculin treatment and F. after treatment. Scale bar 10 µm, false colour showing intensity (colour bar min to max). G. LDCVs at the plasma membrane of the cell highlighted in e. tracked over 100 frames at 14 Hz. Scale bar 2 µm, Tracks coloured as per colour bar from 0 to 100 frames. H. Vesicle displacement from vesicles tracked across field of view in E. Median (blue line) 0.1 µm for both before and after treatment, mean (cross) 0.14 µm and 0.17 µm for before and after treatment. Outliers shown. I. Equatorial section of a PC12 cell showing F-actin and LDCVs imaged under CLSM and deconvolved. Scale bar 2 µm. J. a merge of both components under TIRF illumination with vesicles identified (grey spheres). Scale bar 2 µm. K. zoom into region of interest showing vesicles in gaps of actin >1 µm. Scale bar 1 µm. L. the distribution of the maximum displacement length of vesicle tracks from 16,944 tracks in 5 PC12 cells over 2 minute recordings, error bars show SEM.

Figure S4 Syntaxin and vesicle organisation at the plasma membrane (related to Figure 2 and Figure 3) A dSTORM image map showing the nano-scale positions of immuno-detected (magenta) syntaxin1 molecules in a region of plasma membrane in wild-type (*left* panel) and munc18-1/2 DKD cells (*right*), with the positions of LDCVs shown in green. Scale bars: 1 µm.

Table S1 *in silico* model parameters are predictive
6 different measures of LDCV behavior are presented, with the difference between *in silico* and real control *versus* mutant cells (related to Figure 3).

	Max Dist from EXY	E Dist from XY0	Max Dist XY0	MSD from EXY	MSD from XY0
	0.80 ± 0.04	0.69 ± 0.003	1.09 ± 0.03	0.08± 0.008	0.60 ± 0.01
Control – Mutant distance	**0.80**	**0.69**	**1.09**	**0.08**	**0.61**
	0.20 ± 0.02	0.12 ± 0.02	0.25 ±	0.01 ± 0.007	0.06 ± 0.06

			0.03		
Control – Mutant distance	**0.19**	**0.12**	**0.25**	**0.01**	**0.06**

Explanation of terms:

Max dist from EXY = maximum distance from the mean position
E dist from XY0 = average distance from the starting position
Max dist from XY0 = maximum distance from the starting position
MSD from EXY = mean squared distance from mean position.
MSD from XY0 = mean squared distance from starting position

All units are microns.

In all cases, the sign of the difference between control versus mutant values is the same (i.e. positive in these experiments), indicating the predictive nature of the in silico model. The positive sign here predicted that the vesicles in the mutant cells would become more mobile, as observed.

Supplemental Experimental Procedures

Cell culture, plasmids and transfections

Munc18-1 siRNA PC-12 cells (DKD and KD43) were a kind gift of Shuzo Sugita[S1] and grown in DMEM medium supplemented with 5% (v/v) horse serum, 5% (v/v) fetal bovine serum, 1% (v/v) Penicillin/Strep (Invitrogen), 400 µg/ml G418, 2. 5 µg/ml puromycin and maintained at 37 °C in 7.5% (v/v) CO_2, 92.5% (v/v) air. LDCVs were labelled using NPY-EGFP as previously described[S2]. Transfections were performed using Lipofectamine 2000 (Invitrogen). Where appropriate, cells were stimulated to secrete with 300 µM ATP, added to the bath after 500 frames imaging at 20 Hz, with imaging continued for 1000 further frames. Secretion was visualised using TIRFM as we have described before[S3], by observing ‘flashes’ as lumenal, acidic NPY-EGFP is exposed to the higher, extracellular pH. Lifeact-EGFP and NPY-mCherry were used for F-actin experiments, Latrunculin-A at 15 µM was added to the cells whilst imaging to disrupt actin.

Single molecule imaging

Cells were transfected with either Munc18-1, SNAP-25 or syntaxin1a, fused to PAmCherry and fixed with 4% (w/v) paraformaldehyde, 0.1% (w/v) glutaraldehyde for 90 minutes. PALM acquisition involved cycles of brief activation at 405 nm followed by rapid imaging in TIRF mode at 561 nm. All PALM and dSTORM experiments were performed using an Olympus IX-81 microscope equipped with Olympus Cell^R acquisition software, an ImageEM EM-CCD 512x512 camera (Hamamatsu UK) and an Olympus 150X UAPO 1.45NA oil lens with a resulting pixel size of 106 nm. For dSTORM, endogenous proteins were immunolabelled, after 90-minute fixation in 4% (w/v) buffered paraformaldehyde with primary antibodies (syntaxin1a (HPC-1)). Immunodetected syntaxin1a molecules were subsequently labelled with Alexa-647-conjugated anti-IgG (Invitrogen). Alexa-647 was found to have the greatest propensity to enter a dark state using 640 nm illumination in an oxygen-depleting buffer (50 mM MEA in PBS buffered at pH 7.4).

For sptPALM, the image acquisition regime was similar to above, except live cells were used. Imaging was performed at 37°C in 7.5% (v/v) CO_2 with brief activation pulses at 405 nm followed by trains of 100 frames with 561 nm excitation, with a 30 ms exposure time.

Single molecule detection and tracking was performed essentially as we previously described[S4].

The performance metrics were calculated to have equivalency with the values determined in [S5] from synthetic image data used in [S6].
The signal to noise ratio of the datasets were calculated using an intensity based method; $SNR = (I_0 - I_b)/\sqrt{I_0}$, where I_0 and I_b denote the peak object intensity and the mean background intensity respectively as in [S6].

Confocal imaging

Confocal laser-scanning microscope images were acquired at Nyquist sampling rates using a Leica SP5-SMD inverted system, equipped with a white-light super-continuum laser excitation source using a 63X 1.2NA water immersion lens. EGFP and mCherry were excited at 490 nm and 560 nm, respectively, and emission collected between 500-550 nm and 590 – 670 nm. Image data were subsequently deconvolved using Huygens Pro software (SVI, NL), using a theoretical point-spread-function. Image data were visualised using Imaris (Andor, UK).

Fluorescence Correlation Spectroscopy

Prior to each FCS experiment the values for *Kappa* (the ratio of the axial and waist excitation spot dimensions) and V_{eff} were determined using 10 nM Atto488 and Atto561 (Atto-Tec GmbH, Germany) standards in water at either 25°C or 37°C (Supplemental Figure 3). Atto488 has a well-established diffusion rate of 400 $\mu m^2 s^{-1}$ (Picoquant). The resulting Veff and K were verified using 10 nM purified EGFP and mCherry proteins (25kDa) and 10 nM fused EGFP-mCherry protein (50 kDa) in 150 mM NaCl, 20 mM Tris pH7.4, 1 mM DTT and 0.1% (v/v) Tween 20. The resulting diffusion coefficients of the fluorescent proteins (EGFP and mCherry of 124 ± 0.17 and 106 ± 0.12 $\mu m^2 s^{-1}$ at 37°C respectively n=10) are consistent with Stokes-Einstein (where diffusion is inversely proportional to the hydrodynamic radius of the molecule and the viscosity of the surrounding media) estimated diffusion coefficients of 25 kDa proteins under these conditions. Calibration recordings of 30 seconds were made for each standard. These calibrations determined that the effective volume of the FCS spot was 0.29 ± 0.04 μm^3 at 37°C; all *in cellulo* FCS measurements were made at this temperature.

FCS Analyses

Autocorrelation traces were generated from the photon counting histograms for each 5 to 30 s measurement using SymPhoTime v5.4.4 software (Picoquant GmbH, Germany). *In vitro* calibration traces were fitted using the Triplet model (3D free diffusion model with triplet state) with informed diffusion values to yield Veff and Kappa values. Neuronal autocorrelation traces were fitted using a Triplet Extended model (2-D anomalous diffusion model with triplet state), this model is designed for fluorescent molecules moving within a plane e.g. proteins in a membrane. Diffusion within cells is expected to be anomalous therefore the anomaly parameter was not fixed to one. The anomaly parameter (α) measures the departure from free Brownian diffusion (α =1) to either super-diffusion (α >1) or subdiffusion (α <1) for a diffusing species. Autocorrelation curves with (α >1) display the sharpest decay, whereas the those with α <1 decrease quite slowly. The goodness-of-fit was examined by inspection of the residuals, which should be randomly distributed around zero.

Mathematical modelling

We consider the movement of vesicles in a potential field V formed by the presence of molecules such as SNARE/munc-18. We use for this a classic Langevin type equation that models a large particle immersed in a fluid of smaller ones given by

$$dq = p\, dt$$
$$dp = (B\, p - \alpha\, \mathrm{grad}(V(q)))\, dt + \sigma dW$$

We use a classic Langevin equation that models a large particle (assumed here to have unit mass) in a fluid;
B=-0.82 [1/s], alpha=5 [mu Kg/s^2], sigma=0.6 [Kg mu / s sqrt(s)]
B=-0.82 [1/s], alpha=1.25 [mu Kg/s^2], sigma=0.6 [Kg mu / s sqrt(s)]
mu=micro meters.
q=(q_1, q_2)^T is the displacement and where alpha is the weight of the forcing. The term Bp represents a friction term (since B is taken to be negative) and σdW models the random collisions with smaller molecules in the cell. W is a standard Brownian motion.

We construct the potential field V from real biological data on the positions of molecules in a particular cell imaged using PALM. At each molecule position we have the peak of a two dimensional Gaussian function centred on this position. Other forms of potential were also investigated but the Gaussian was kept for simplicity. The field is scaled so that max(V(q)) =1 and min(V(q))=0 and the radius of the molecule (taken to be 5e-3 microns) was matched to a potential of approximately > 0.9. This yields a potential field such as shown, for example, in Figure 2. We do not impose boundary conditions on the computational domain, instead, once a vesicle leaves the computational domain its track is no longer considered. Initial data for the vesicle positions q(0) were determined from corresponding PALM data and all synthetic vesicles were started with an initial velocity of zero.

Numerical implementation.

Given the potential field V it remains to find appropriate parameters for the friction term B, the strength of the stochastic forcing σ and weight of the potential α. For the control data set we take: B=-0.82, α =5 and σ = 0.6 whereas for the mutant data set we take B=-0.82, α =1.25 and σ = 0.6. That is we assume the same friction, same noise term and simply reduce the strength of potential field (and hence influence of the molecules) by 1/4. Initially a least-squares fit to the observed mean(log(average area)) was examined for the control data this leads to different parameter values (B=-1.18, α = 9.59, σ = 0.6), however the variance around the mean was not realistic. Here average area refers to the maximum distance travelled by the vesicle from its average position within its track, scaled by tracklength. The parameter values actually used were then determined by hand for the control. For the mutant the single parameter α was used to obtain the results. This gives weight to the idea that a change in the potential field of surrounding molecules is a key difference between the mutant and control.

In implementation a spline interpolant was used for second order finite difference approximations of the derivatives of potential V(q) as this proved computationally more efficient. Statistics on track length and distance are determined

using the same time scales T0 between frames for the experimental data (50x1e-3 s for the control and 70x1e-3 for the mutant) and the final time solved for was the same as in the experiments (corresponding to 500 and 358 frames respectively). However, the tracks of the modelled vesicles are resolved at smaller time scale than is feasible in the experiments and in both cases we took T0/40 as our numerical step size. Standard (explicit and semi-implicit) Euler-Maruyama type schemes were used to integrate the Langevin equations. We examined 100 vesicles arriving at six different initial positions in the computational domain leading to 600 vesicles for each realization of the cell. The simulation was then performed 4 times to compute statistics.

Overlap. The potential field V for the molecules may be interpreted as a probability field for having a molecule at a particular position – thus if V(q)>0.9 it is highly likely that a molecule would be found. We can also construct a probability field for the positions of the vesicles with an in-plane radius of 82.5 microns. We can then consider the probability of an overlap between the molecules and the vesicles by examining the area covered by the molecules and vesicles and by subtracting.
For cellular data, we examined the probability the vesicles and molecules do not overlap. This was done for squares inside the cell with different side lengths (e.g. 1,2,3,4,5,6 microns). In total we take 7 sizes on two different parts of each of the cells to get an average chance of not overlapping (as in Figure 2).

Mean-squared displacement (MSD) is typically used to identify if displacement from a fixed (typically initial) position grows linearly in time, indicating that a particle is undergoing Brownian motion, looking at a distance (RMSD) travelled then simply changes the scaling. We have introduced in this article novel ideas for examining the time series data of particle movement. In Fig. 3 we look at "Average Area" which captures how far the vesicle moves from its mean position i.e. does it travel a long distance or a short distance from where you expect it to be.

Statistical methods
Statistical analyses were performed using GraphPad Prism. Data sets were first tested for normality using the Shapiro-Wilks test. Data that fitted a normal distribution was tested for statistical significance by two-tailed unpaired Students t-test. Failing Normality, the data were analysed using the Mann-Whitney test. All histogram data are presented as standard deviation of the mean (±S.D.) and boxplot data as min and max data, for completeness. Boxplots were created using BoxPlotR tools [S8]. We estimated the statistical power of our conclusions where appropriate and for vesicle and single-molecule dynamic comparisons, found all datasets to provide a power of >99% at the replicate number we employed using a post-hoc power calculation[S9]. For the data in Figure 3d, the data were tested for Normality using the Shapiro-Wilks test, then a 2-way ANOVA with the Kuskal-Wallace test.

Supplemental references

S1. Arunachalam, L., Han, L., Tassew, N. G., He, Y., Wang, L., Xie, L., Fujita, Y., Kwan, E., Davletov, B., Monnier, P. P., et al. (2008). Munc18-1 Is Critical for Plasma Membrane Localization of Syntaxin1 but Not of SNAP-25 in PC12 Cells. Mol Biol Cell *19*, 722–734.

S2. Rickman, C., Medine, C. N., Bergmann, A., and Duncan, R. R. (2007). Functionally and spatially distinct modes of munc18-syntaxin 1 interaction. J. Biol. Chem. *282*, 12097–103.

S3. Smyth, A. M., Rickman, C., and Duncan, R. R. (2010). Vesicle fusion probability is determined by the specific interactions of munc18. J. Biol. Chem. *285*, 38141–8.

S4. Yang, L., Dun, A. R., Martin, K. J., Qiu, Z., Dunn, A., Lord, G. J., Lu, W., Duncan, R. R., and Rickman, C. (2012). Secretory Vesicles Are Preferentially Targeted to Areas of Low Molecular SNARE Density. PLoS One *7*, e49514.

S5. Wilson, R. S., Yang, L., Dun, A., Smyth, A. M., Duncan, R. R., Rickman, C., and Lu, W. (2016). Automated single particle detection and tracking for large microscopy datasets. R. Soc. open Sci. *3*, 160225.

S6. Chenouard, N., Smal, I., de Chaumont, F., Maška, M., Sbalzarini, I. F., Gong, Y., Cardinale, J., Carthel, C., Coraluppi, S., Winter, M., et al. (2014). Objective comparison of particle tracking methods. Nat. Methods *11*, 281–9.

S7. Wilhelm, B. G., Mandad, S., Truckenbrodt, S., Kröhnert, K., Schäfer, C., Rammner, B., Koo, S. J., Claßen, G. A., Krauss, M., Haucke, V., et al. (2014). Composition of isolated synaptic boutons reveals the amounts of vesicle trafficking proteins. Science *344*, 1023–8.

S8. Spitzer, M., Wildenhain, J., Rappsilber, J., and Tyers, M. (2014). BoxPlotR: a web tool for generation of box plots. Nat. Methods *11*, 121–2.

S9. Levine, M., and Ensom, M. H. (2001). Post hoc power analysis: an idea whose time has passed? Pharmacotherapy *21*, 405–9.
