# Supplementary figures and images for: Navigation through the Plasma Membrane Molecular Landscape Shapes Random Organelle Movement
Source: Curr Biol. 2017 Feb 6;27(3):408–14. doi: 10.1016/j.cub.2016.12.002 (PMC5300901; doi:10.1016/j.cub.2016.12.002)

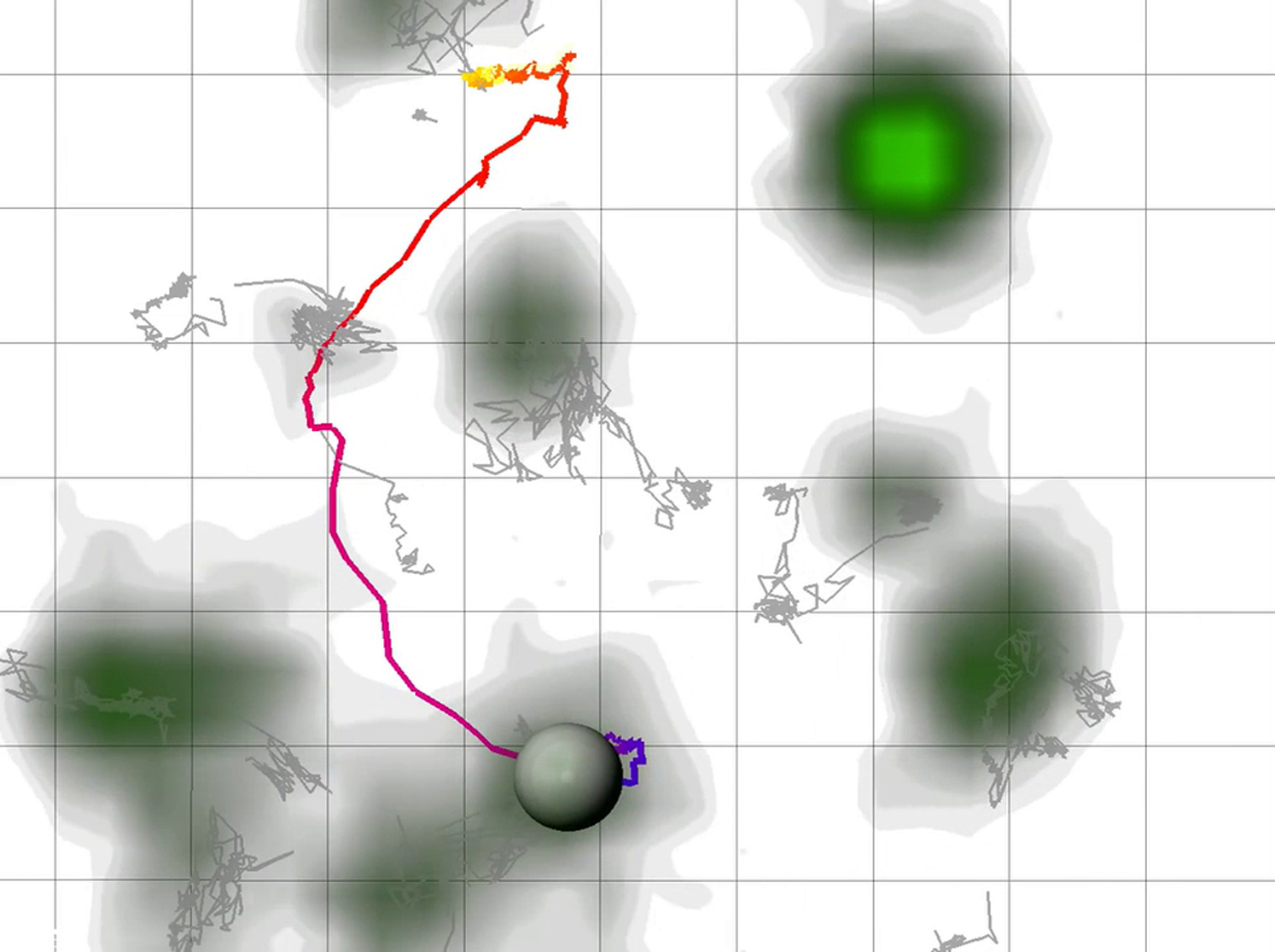

Supplement: Movie S1. A Mobile Vesicle, Related to Figure 1B — A mobile vesicle (gray sphere and colored track) scanning the membrane over sites previously visited by other vesicles (gray tracks). Grid scale: 500 nm grid edge, color scale shows 0–28 s, frame rate: 40 Hz. [file mmc2.jpg]

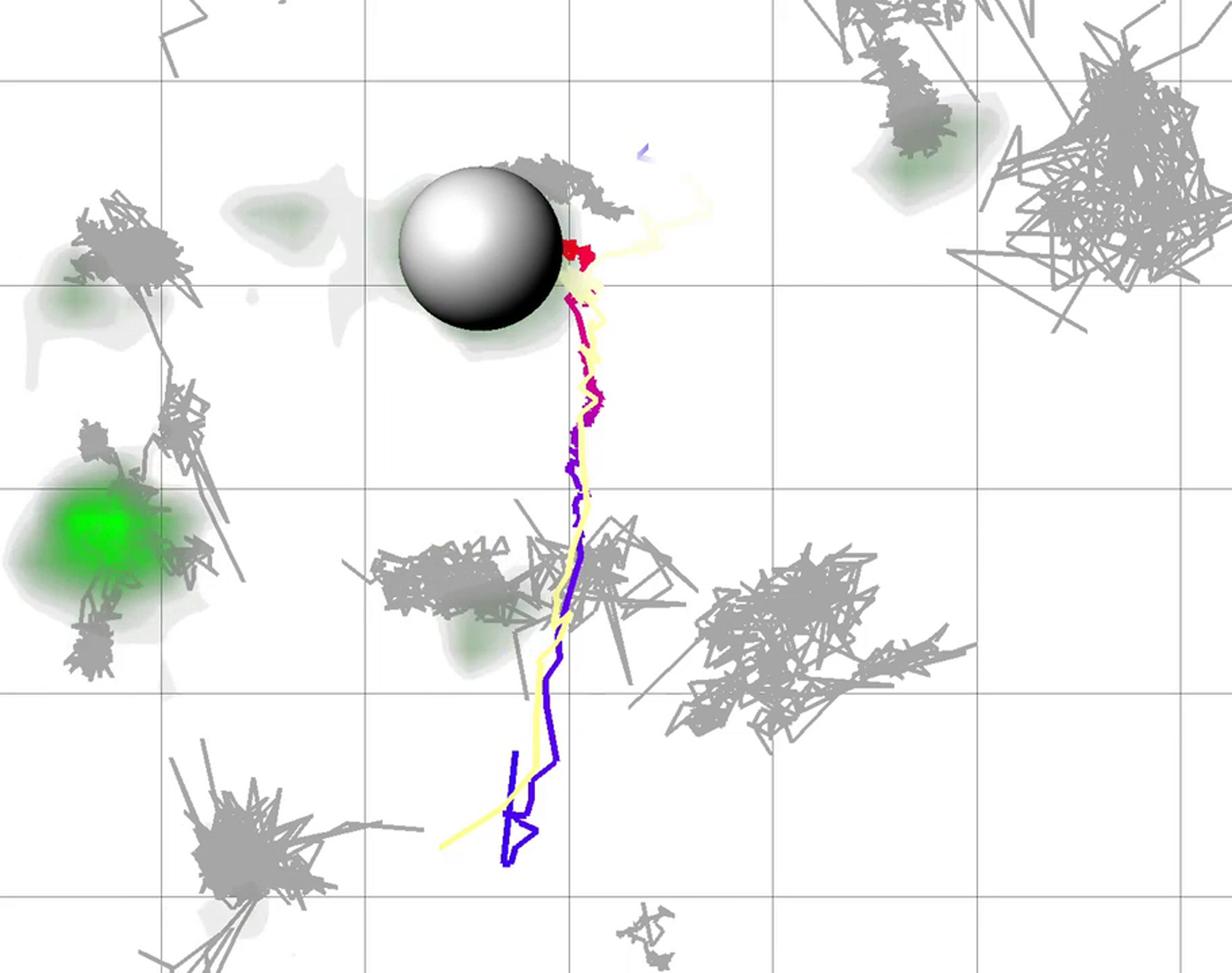

Supplement: Movie S2. Newly Recruited Vesicles Arriving at the Plasma Membrane, Related to Figure 3 — Newly recruited vesicles arriving at the plasma membrane (gray sphere, colored track) and moving along similar paths to similar fusion sites. Previous vesicle tracks (gray), grid scale: 500 nm grid edge, color scale shows 0–41 s, frame rate: 40 Hz. [file mmc3.jpg]

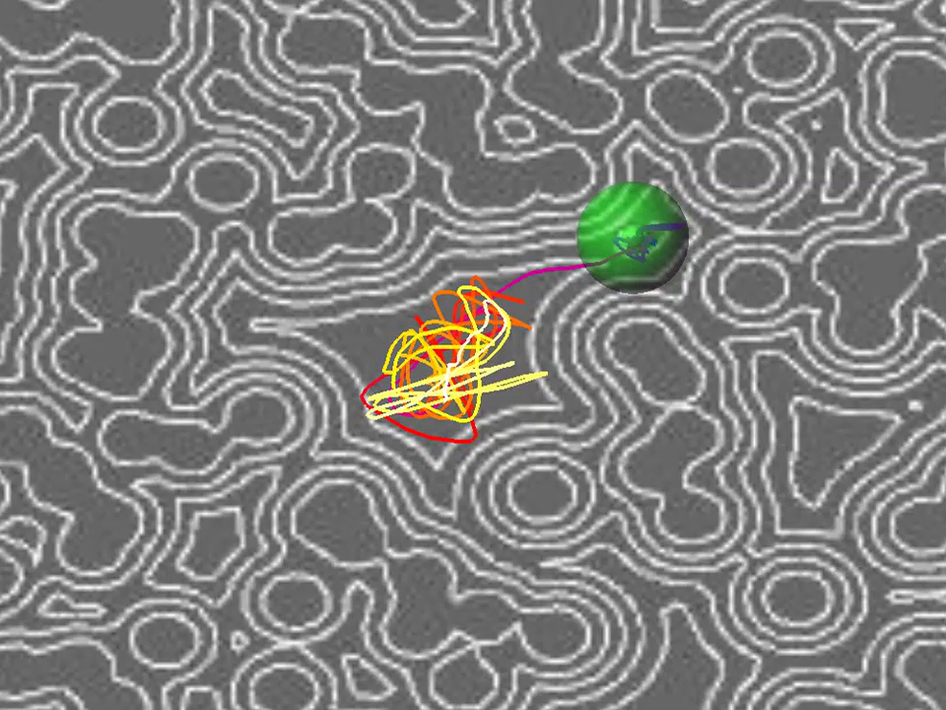

Supplement: Movie S3. A Typical In Silico Vesicle, Related to Figure 3 — The large green circle is 400 nm in diameter and represents the LDCV diameter. The smaller circle inside this is 165 nm diameter and represents the meaningful “functional” diameter of an LDCV that can sample the membrane molecular landscape (described in [S4]). Gray and white contours represent molecular mountains as shown in Figure 3, color scale shows 0–24 s, frame rate: 40 Hz. [file mmc4.jpg]
