# Supplementary material for: Navigation through the Plasma Membrane Molecular Landscape Shapes Random Organelle Movement
Source: Curr Biol. 2017 Feb 6;27(3):408–14. doi: 10.1016/j.cub.2016.12.002 (PMC5300901; doi:10.1016/j.cub.2016.12.002)
Supplement: Document S2. Article plus Supplemental Information [file mmc5.pdf]

Current Biology

Report

Navigation through the Plasma Membrane Molecular Landscape Shapes Random Organelle Movement

Graphical Abstract

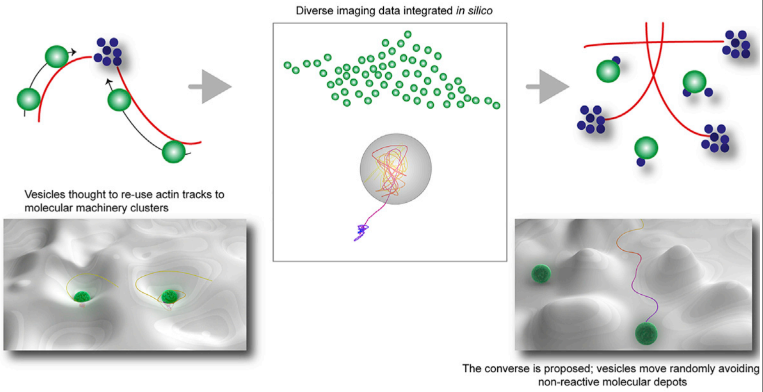

Authors

Alison R. Dun, Gabriel J. Lord,
Rhodri S. Wilson, ...,
Andreas Papadopulos, Colin Rickman,
Rory R. Duncan

Correspondence

r.r.duncan@hw.ac.uk

In Brief

Dun et al. use mathematical modeling, super-resolution imaging, and biological perturbations to shape the membrane molecular landscape, examining the inter-relationship between the plasma membrane and organelle dynamics. They show that secretory vesicles at the cell surface move randomly, navigating among depots of secretory machinery molecules.

Highlights

- Membrane-proximal vesicle movements are random
- Secretory molecules at the plasma membrane organize into conformational clusters
- Random vesicle movements navigate among these clusters
- This model explains vesicle behaviors without a requirement for functional pools

Dun et al., 2017, Current Biology *27*, 408–414
February 6, 2017 © 2016 The Author(s). Published by Elsevier Ltd.
http://dx.doi.org/10.1016/j.cub.2016.12.002

CrossMark
CellPress

CellPress

Current Biology
Report

Navigation through the Plasma Membrane Molecular Landscape Shapes Random Organelle Movement

Alison R. Dun,[1,2] Gabriel J. Lord,[3] Rhodri S. Wilson,[1,2] Deirdre M. Kavanagh,[1,2] Katarzyna I. Cialowicz,[1,2] Shuzo Sugita,[4] Seungmee Park,[4] Lei Yang,[1,2,7] Annya M. Smyth,[5] Andreas Papadopulos,[6] Colin Rickman,[1,2] and Rory R. Duncan[1,2,8,*]
[1]Institute of Biological Chemistry, Biophysics and Bioengineering, Heriot-Watt University, Edinburgh EH14 4AS, UK
[2]Edinburgh Super-Resolution Imaging Consortium
[3]Department of Mathematics, Maxwell Institute, MACS, Heriot-Watt University, Edinburgh EH14 4AS, UK
[4]Toronto Western Research Institute, Room 11-432, McLaughlin Wing, 399 Bathurst St., Toronto, ON M5T 2S8, Canada
[5]Centre for Inflammation Research, University of Edinburgh, The Queen's Medical Research Institute, 47 Little France Crescent, Edinburgh EH16 4TJ, UK
[6]The Clem Jones Centre for Ageing Dementia Research, Queensland Brain Institute, The University of Queensland, Brisbane, QLD 4072, Australia
[7]Present address: FutureWei Technologies (Huawei R&D), Santa Clara, CA 95050, USA
[8]Lead Contact
*Correspondence: r.r.duncan@hw.ac.uk
http://dx.doi.org/10.1016/j.cub.2016.12.002

SUMMARY

Eukaryotic plasma membrane organization theory has long been controversial, in part due to a dearth of suitably high-resolution techniques to probe molecular architecture in situ and integrate information from diverse data streams [1]. Notably, clustered patterning of membrane proteins is a commonly conserved feature across diverse protein families (reviewed in [2]), including the SNAREs [3], SM proteins [4, 5], ion channels [6, 7], and receptors (e.g., [8]). Much effort has gone into analyzing the behavior of secretory organelles [9–13], and understanding the relationship between the membrane and proximal organelles [4, 5, 12, 14] is an essential goal for cell biology as broad concepts or rules may be established. Here we explore the generally accepted model that vesicles at the plasmalemma are guided by cytoskeletal tracks to specific sites on the membrane that have clustered molecular machinery for secretion [15], organized in part by the local lipid composition [16]. To increase our understanding of these fundamental processes, we integrated nanoscopy and spectroscopy of the secretory machinery with organelle tracking data in a mathematical model, iterating with knockdown cell models. We find that repeated routes followed by successive vesicles, the re-use of similar fusion sites, and the apparently distinct vesicle "pools" are all fashioned by the Brownian behavior of organelles overlaid on navigation between non-reactive secretory protein molecular depots patterned at the plasma membrane.

RESULTS AND DISCUSSION

To address the question of whether membrane-proximal vesicles behave in a controlled manner, we first posed a simple question: when new vesicles are recruited to the plasma membrane, is this spatially random, or is there some order to this process? We labeled large dense-core vesicles (LDCVs) in secretory cells (phaeochromocytoma cells, PC12s) using soluble cargo Neuropeptide Y (NPY) fused to EGFP [17, 18]. We stimulated cells to secrete and then followed the recruitment of new LDCVs using total internal reflection fluorescence microscopy (TIRFM). We quantified trajectories taken by all vesicles before, during, and after exocytosis. This marked the image plane of the plasma membrane with areas visited by LDCVs during the recording period and allowed us to determine the arrival sites of any new recruits. Figure 1B displays frames from Movie S1, showing a vesicle arriving on top of a site occupied by an earlier LDCV, scanning the same region before moving off to visit at least two other previously occupied regions. The area under scrutiny is ~4 μm^2. We found LDCVs follow similar trajectories to similar (but not identical) fusion sites on the cell surface (Figures S2A–S2D), even after treatment with Methyl-β-cyclodextrin to quantifiably disrupt plasma membrane lipid order [19] (Figures S2E–S2G). These data appear to support current models, suggesting that LDCVs visit preferred sites on the membrane, using defined, re-usable routes that intuitively appear like physical tracks.

We previously used photoactivated localization microscopy (PALM) [20, 21] and direct stochastic optical reconstruction microscopy (dSTORM) [22] to relate syntaxin1a and SNAP-25 molecular positions with those of single LDCVs [4, 5, 18]. We examined mCherry molecule aggregation in living cells, to exclude the possibility that the non-uniform patterning of the SNARE fusions at the plasma membrane could be caused by fluorescent protein oligomerization. For this we used fluorescence correlation spectroscopy, as we have before [4], in primary cells and cell lines, finding no evidence of aggregation of fusions or unfused mCherry.

We found previously that the number of tSNARE/SM molecules residing within functionally relevant distances of LDCVs near the plasma membrane is very low, with LDCVs encountering small numbers of isolated tSNAREs/SM proteins located between molecular clusters or depots. This was confirmed using stimulated emission deletion (microscopy; STED) imaging of

 © 2016 The Author(s). Published by Elsevier Ltd.
This is an open access article under the CC BY license (http://creativecommons.org/licenses/by/4.0/).

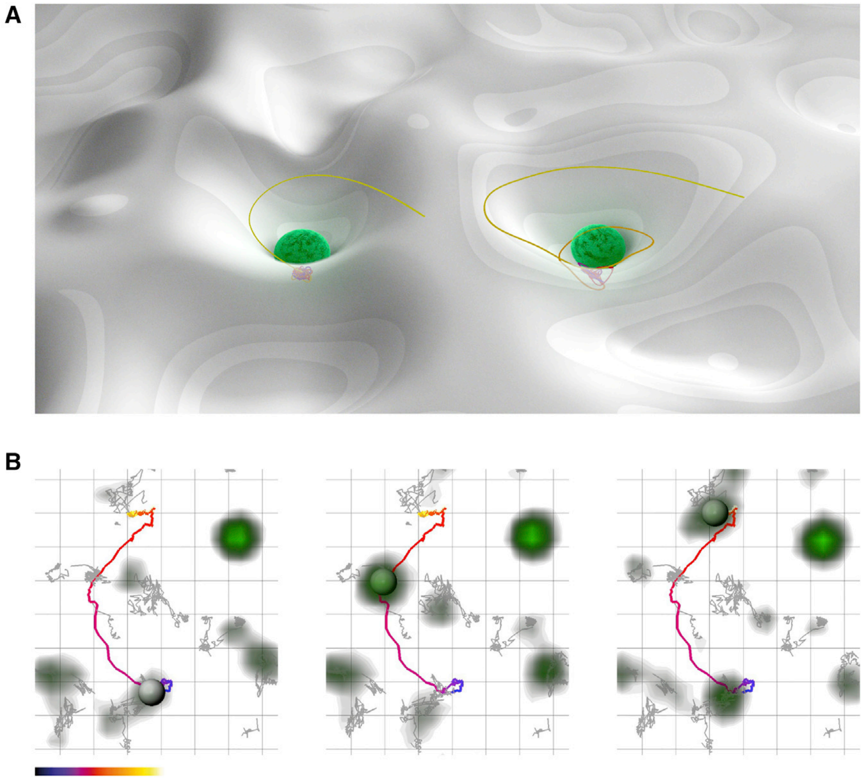

Figure 1. Current Model for Vesicle Dynamics at the Membrane

(A) Stylized cartoon showing a model for vesicle dynamics at the plasma membrane, where LDCVs are docked on molecular machinery depots. This is illustrated as a "well," attracting LDCVs.

(B) Images from a TIRFM recording of a PC12 cell expressing lumenal NPY-EGFP. A single vesicle (gray sphere) is shown (track in color) scanning the membrane, visiting areas preferred by other vesicles (gray tracks). Grid scale, 500 nm grid edge. Color bar shows time.

See also Figure S1 and Movie S1.

endogenous SNAREs, finding immuno-labeled LDCVs surrounded by tSNARE clusters [17]. Similar results were found for the SM protein and syntaxin1a chaperone munc18-1 [5], marrying imaging with biophysics techniques that showed small numbers of SNARE/SM molecules are required to drive vesicle fusion.

We created density maps of the cell surface from vesicle dynamics data, finding that the pattern of vesicle trajectories was non-uniform over the cell surface, with many sites connected by common tracks, visited by multiple vesicles. This presented a conundrum: if LDCVs move along re-usable paths, revisiting specific membrane sites, what do these apparent routes connect, if not SNARE/SM depots?

To address this, we modeled data in silico, integrating quantitative information describing molecular numbers, positions, and densities with dynamic datasets describing similar parameters for LDCVs, iterating with knockdown cells to perturb the biological system and compare with predictions made by our mathematical model. We considered vesicles in a potential field formed by tSNARE and SM proteins. Munc18-1 is present at the membrane only by virtue of a 1:1 stoichiometry interaction with the tSNARE, syntaxin1, or the tSNARE heterodimer [4], so the potential fields were constructed from peaks of a two-dimensional Gaussian function centered on each molecular coordinate describing munc18-1 location. This was informed using PALM data from munc18-1-null cells, rescued functionally by PA-mCherry-munc18-1 [5] (Figures 2A and 2B). The model is deliberately simple, but it displays key qualitative and quantitative features that indicate it might be a good match to the biological data. Our aim was not a precisely calibrated model, but rather to show that this standard incomplete model can explain the observed behavior of vesicles in living cells as well as predict biological outcomes following perturbations.

Vesicle positions determined from corresponding TIRFM data were added, incorporating information about vesicle dynamics from those experiments; i.e., arrival position, movement, speed, etc. We further informed our model with a probability field for the positions of the vesicles with an in-plane radius of 82.5 nm, as we explained before [18]. This represents a distance from the inner leaflet of the plasma membrane where membrane-proximal LDCVs can interact with tSNARE molecules. We then considered the probability of functional overlap between the molecules and the vesicles, modeling real data and examining the probability that vesicles and molecules meet (Figures 2C and 2D). This supported the imaging experiments, indicating a very low probability of LDCV and tSNARE/SM molecule interaction; importantly, at diffraction-limited resolution, "colocalization" is common, but this cannot be functionally relevant [18].

We questioned whether LDCVs move along actin filaments, as sometimes suggested. To do this, we acquired dual-color TIRFM images from living cells expressing Lifeact [23]-EGFP and NPY-mCherry. These experiments showed no correlation between actin features and vesicle trajectories (Figures S3A–S3D). We perturbed the actin cytoskeleton using Latrunculin A, resulting in partial rearrangements in Lifeact-labeled structures. We found no effect of this intervention on the vesicle behaviors we measured (Figures S3E–S3H). To investigate this further, we used confocal laser-scanning microscopy (CLSM), visualizing the actin cortical layer, LDCVs, and the plasma membrane in the same samples. Combined with image data deconvolution, this provides lateral resolution of around 200 nm, sufficient to resolve potentially separate arrangements of these structures. Equatorial sections in these images confirmed that LDCVs near the cell surface appear embedded within the cortical layer (as reviewed in [24]), but, importantly, vesicles there reside in "spaces" in the actin network (Figure S3I). This is impossible to assess in CLSM equatorial sections, so we next performed TIRFM on the same samples. This analysis revealed that, indeed, membrane-proximal LDCVs reside in windows in the cortical

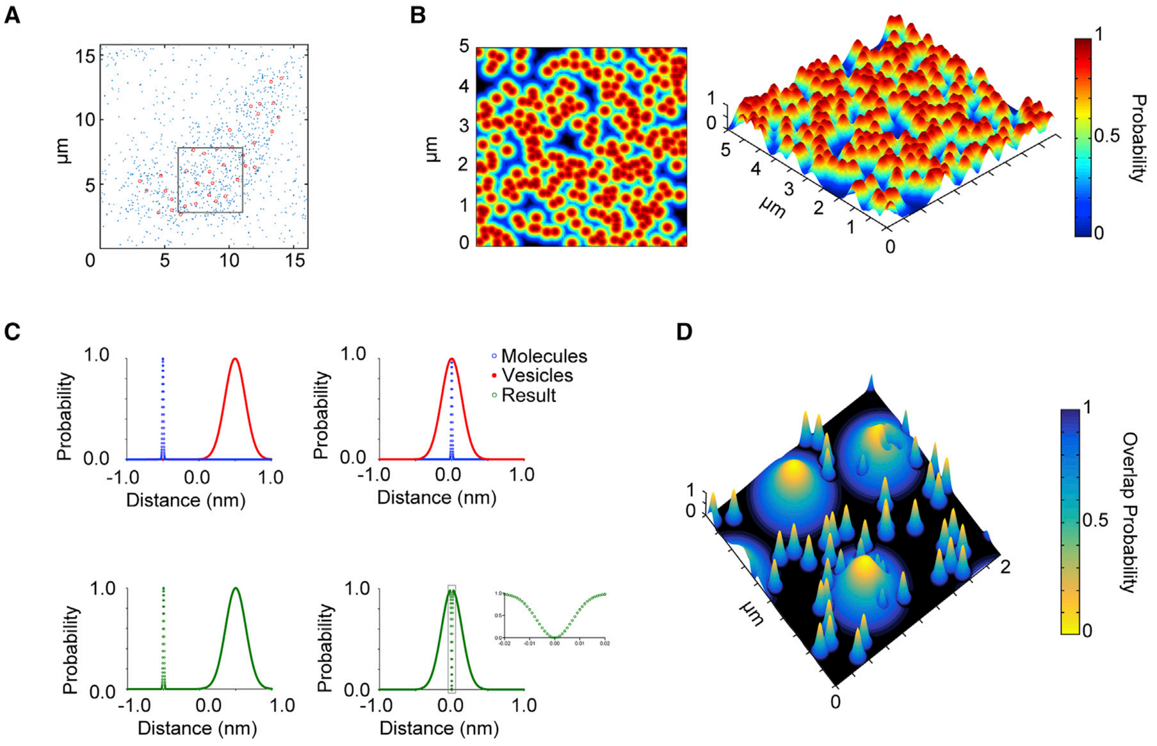

Figure 2. Probability of Syntaxin1a Molecule/LDCV Functional Overlap Is Low

(A) Molecule and vesicle positions derived from real data, with molecules represented in blue and vesicles in red.

(B) Molecular densities plotted for a 5 μm^2 region of plasma membrane, illustrating the probability of molecules being present, represented in 2D (left) and 3D (right) plots.

(C) A method to measure overlap is shown graphically, using synthetic data, with molecules in red, vesicles in blue, and the result of combining the two in green, where the probability of a molecule localization is subtracted from the probability of a vesicle position (described in the Supplemental Experimental Procedures).

(D) Vesicles are predominantly in molecular gaps and overlap probability with SNARE/SM depots is low.

See also Figures S2 and S4.

actin network that are larger than the longest walks taken by membrane-proximal vesicles. Together, these experiments show directly that LDCVs at the membrane do not follow actin fibers, nor are they constrained necessarily by the cortical network there (Figures S3J–S3L).

Thus, we re-visited the current understanding of vesicle dynamics. We attempted to replicate the use of repeated routes connecting tSNARE/SM depots by LDCVs (Figure 1; Movies S1, S2, and S3) in silico by creating "potential wells" defining tSNARE/SM protein positions and densities, attracting modeled vesicles. Biologically, this could be explained by vesicles being directed to sites of molecular interaction, representing most current understanding.

In agreement, this rehearsal of the mathematical model did not reiterate the biology (nor did it represent the molecule/vesicle probability overlap revealed by nanoscopy). Instead, we turned this problem around and asked what would happen if the protein depots were *avoided* by the model vesicles.

We computed the dynamic behavior of 600 virtual vesicles in four replicates, starting with the initial positions and numbers of vesicles and molecules observed in the real biological data. In this scenario, the in silico vesicle behavior reiterated the real biology rather well, with vesicles navigating along "valleys" between the molecular densities, buffeted in an otherwise Brownian manner (Figures 3A and 3B). This finding was encouraging, providing an alternative description for dynamics seen at the cell surface; no physical tracks need be present to explain LDCV saltatory movements, changing speeds, the apparent re-use of trajectories, or re-sampling of membrane sites. Using this theory, all vesicle behaviors can be described with a single continuum (Figure 3C).

We expected that if exocytotic protein machinery depots were depleted from the cell surface, so the molecular "valleys" that shape the LDCV trajectories would also be altered; in this case, LDCV movements should shift toward Brownian behavior. Disrupting munc18-1 expression, while invaluable [26], has been problematic for two reasons; first, munc18-1 and syntaxin1 expression levels, functions, and localizations are intimately linked, meaning that disrupting one affects the other [27]. Second, munc18-2 can compensate for munc18-1 in cells and in null animals [28]. We therefore used munc18-1/2 double knockdown (DKD) cells shown to lack munc18 function [28]. Munc18-1, syntaxin1a, and syntaxin1a/SNAP-25 heterodimer are all postulated to be "docking factors" in the literature [26, 29, 30], illustrating the inter-linked nature of their biology. Having suggested that LDCVs navigate between tSNARE/SM depots, we wanted to determine whether this was due to the presence of syntaxin1 or Munc18 molecules

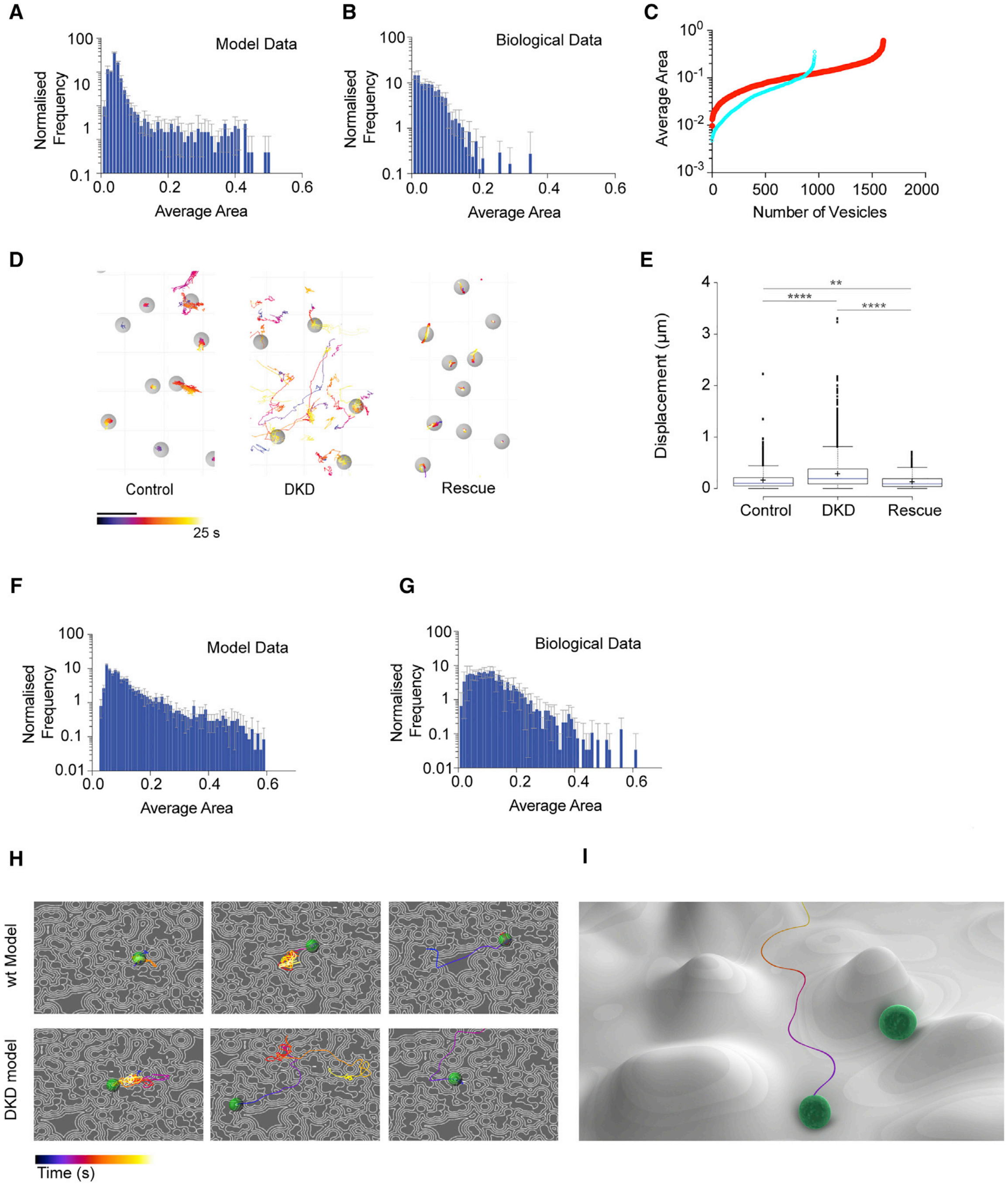

Figure 3. Modeling LDCV Dynamics In Silico Reiterates Biological Data Only if Vesicles Avoid Secretory Machinery Depots

(A) The average area sampled by vesicles, generated from 2,400 in silico vesicles over four iterations.

(B) A similar measurement for real biological data (941 vesicles in four cells) reiterates the mathematical model. Error bars show the SD.

(C) The distribution of real vesicle dynamics follows a continuum with no statistically distinct sub-pools of vesicle behavior (wild-type [WT], blue; mutant [DKD], red).

(D) Images show vesicle tracks within an area of plasma membrane. Left, WT cells; middle, DKD M18 cells; right, DKD M18 cells rescued with munc18-1. Gray spheres (vesicles) are 400 nm diameter. Scale bar, 1 μm. Color scale for tracks spans 25 s.

(E) Boxplots show displacement for all vesicles tracked in WT PC12, DKD M18, and control DKD M18 cells rescued with munc18-1. Line shows median displacement, with outliers presented. Kruskal-Wallis test, ****$p > 0.0001$ and **$p > 0.01$.

(F) The average area sampled by in silico vesicles, generated from our mutant model for 2,400 vesicles from four iterations.

(G) The same average area measurement for real biological data from DKD cells (1,606 vesicles from four cells). Error bars show the SD.

(legend continued on next page)

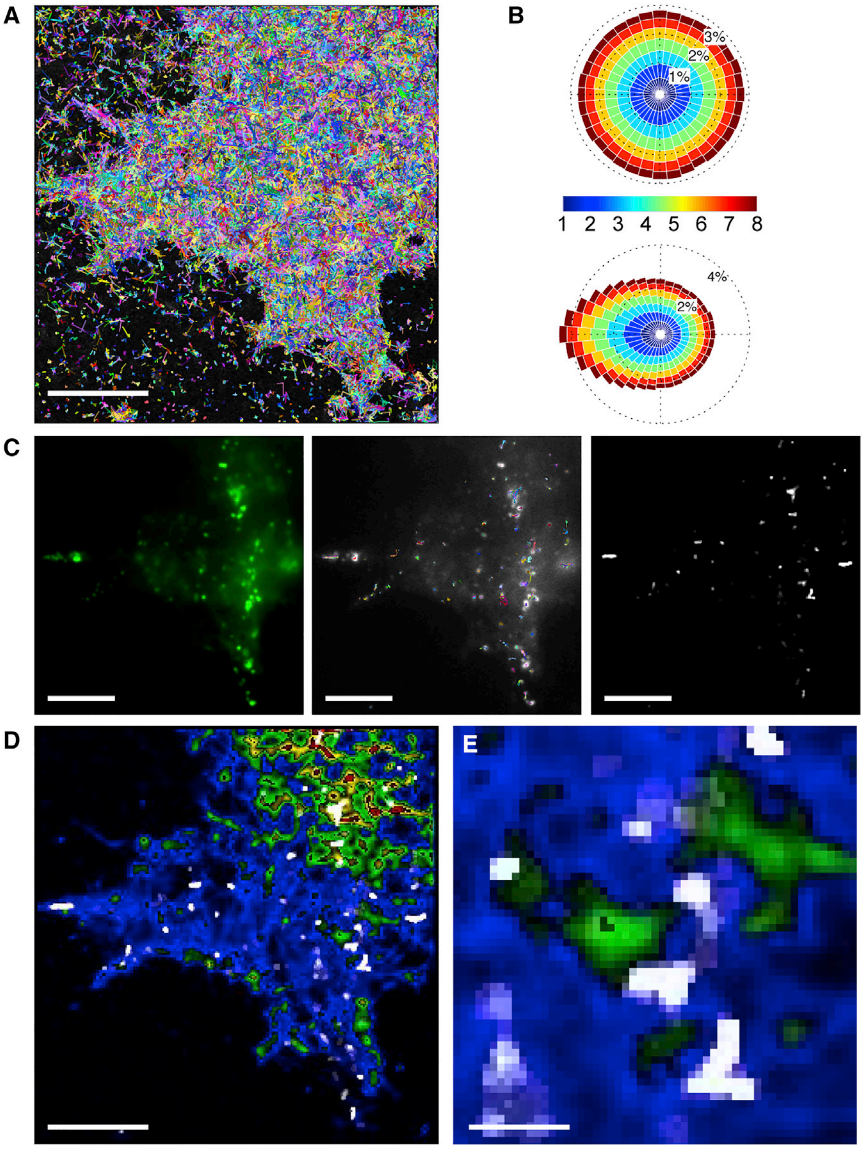

Figure 4. LDCVs in Living Cells Navigate among tSNARE Molecular Depots Containing munc18 Proteins

(A) Single-particle trajectories of 245,964 syntaxin1a molecules in a living cell. Track color is only for contrast.

(B) Rose diagrams are angular histograms with 36 bins, each of 10°. Histogram bin magnitude indicates the number of molecule tracks with that direction relative to normal, and color is molecular speed. This shows that tSNARE molecules behave in a Brownian manner with complete freedom of direction in their initial movements, illustrated as a symmetrical (circular) histogram (top). If a preceding direction is known from tracking data, the next step in molecular direction is frequently a reversal, shown by a skewing to the left in the Rose diagram (bottom).

(C) LDCVs labeled with lumenal NPY-EGFP in the same cell (left). Vesicles were tracked (middle) with track color for contrast. Trajectory information was converted into a density map, representing the number of LDCV tracks crossing each pixel in the image over time (right; white densities).

(D and E) The LDCV track density (D, white) was overlaid on a density map (color) of syntaxin1a molecule tracks, with the boxed area magnified in (E). These experiments show directly that LDCVs navigate paths among tSNARE depots.

Scale bars, 5 μm (A, C, and D) and 1 μm (E). See also Figure S4.

or whether both are required. To look at this, we performed dSTORM, immuno-localizing syntaxin1 molecules that reached the cell surface in munc18-1/2 DKD cells. This revealed that plasma membrane syntaxin1 molecules are present with a similar distribution to that in wild-type cells (Figure S4). Performing vesicle-tracking experiments agreed with our prediction; membrane-proximal vesicles in these cells adopted an increased Brownian motion compared to control cells or to DKD cells rescued with heterologous munc18-1 expression, with longer walks, using unique paths (Figures 3D and 3E). We modified our model parameterization to reduce the magnitude of molecular depots accordingly and found that in silico vesicle dynamics reiterated the real biology (Figures 3G–3I). This echoed the idea that whereas our model is not precisely quantitative, it can predict the effects of specific molecular perturbations on the dynamics of intracellular organelles (Table S1). These experiments suggested that if the equilibrium of reactive versus non-reactive SNAREs is altered (e.g., by depletion of SM proteins), then LDCV behavior is altered in a predictable way (Figure 3J).

Finally, we returned to live-cell imaging to examine directly the vesicle dynamics in relation to tSNARE/SM depots. We acquired data from NPY-EGFP-labeled LDCVs, followed immediately by single-particle tracking-PALM (sptPALM [31]). We used our sptPALM algorithms [4, 5, 18, 32] to provide high-density trajectory maps, super-imposing the LDCV-tracking data onto these. A sample of these data, from four independent experiments, is shown in Figure 4A, displaying 245,964 syntaxin1a-PAmCherry molecular tracks in a single cell. We analyzed the vectors taken

(H) In silico vesicles show a variation in dynamics (suggestive of "immobile" or "scanning" as previously suggested [25]), but with all behaviors falling on the same continuum shown in (C). Top panels show in silico vesicles from the WT model (see also Movie S3), and bottom panels show results when molecular depots are depleted (to mimic DKD cells).

(I) Stylized cartoon showing our model for vesicle dynamics at the plasma membrane, where LDCVs avoid molecular machinery depots. This is illustrated as vesicles following valleys.

See also Figure S3.

by every tSNARE in the dataset, finding that molecules have complete freedom of movement in their initial movements (Figure 4B, top; see also Figure S1), consistent with Brownian diffusion in the membrane plane. We next examined the course followed once a molecule is already moving, finding that the tSNARE molecules behave as if caged in the membrane, with frequent reversals in direction (Figure 4B, bottom; as we previously showed using different techniques and in different cell types in multiple [n > 15] experiments [4, 5, 18]).

Next, we examined LDCV position in the same samples (Figure 4C, left), tracking each single LDCV as before (Figure 4C, middle). We converted tracks into density maps, showing the frequency that an LDCV crossed each pixel in the image (Figure 4C, right). We super-imposed this onto a map describing the density of all tSNARE molecular tracks (Figure 4D), demonstrating that LDCV tracks are contained in the valleys between tSNARE depots, confirming our hypothesis and validating our model.

The combination of data from different imaging modalities remains challenging, as does the interpretation of the data in the biological context beyond simply locational information. Here, we used single-molecule localization coordinate data to inform a mathematical model, integrating information from a variety of imaging and spectroscopic approaches in silico, where such analysis is not possible in the real world.

We propose that clustering of the tSNARE proteins, alongside spatially modulated SM protein sequestration of syntaxin molecules at the membrane [14], creates landscapes of non-reactive plasma membrane [33] interspersed with reactive SNAREs that LDCVs navigate. We have shown before that the tSNARE heterodimer is largely assembled at the plasma membrane [14], that munc18-1 is present at the plasma membrane by virtue of interaction with either monomeric syntaxin1, or the heterodimer [34], and that this interaction is maintained throughout exocytosis [4]. We [17] and others [35] have shown that the composition of the SNARE/SM cluster may vary with spatial location at the cell surface and that these clusters are interspaced with smaller numbers of SNARE and SM proteins [5]. The patterning of SNARE proteins and vesicle recruitment has been shown to be ordered by phosphatidylinositol 4,5-bisphosphate (PIP2) [36]; further work may reveal the non-protein membrane components, such as PIP2, control vesicle dynamics and movements, as well as ‘‘docking.’’ We show little spatial correlation between actin networks and LDCV trajectory; nevertheless, the role of the actin cortical layer in regulating the supply of vesicles to the membrane is clear [12]. Recent work suggested that stable SNARE depots themselves are anchored by the actin cytoskeleton [37]. Our work complements these studies, and we propose that the underlying membrane composition, combined with actin and clustering of membrane proteins, shapes the Brownian motion of membrane-proximal organelles. This theory apparently contrasts with some earlier works [10], probably because of the higher spatial resolutions accessed here that show that ‘‘colocalization’’ cannot correlate with function [5, 18]. Here, we have not looked at fusion sites with molecular resolution, and so it may be that at the site of exocytosis, rapid rearrangements of the secretory machinery occur, as earlier studies suggest [10, 38]. The behavior of secretory vesicles and other organelles at the plasma membrane has never been explained adequately, with current models relying on subjective categorization of apparent behaviors into sub-groups. This in turn correlates with models describing different stages of the secretory pathway that are also not entirely satisfactory. The non-uniform spatial and functional patterning of tSNARE molecules at the plasma membrane, organized by the underlying lipid composition, has been shown to be essential for neurotransmission by guiding synaptic vesicles to active membrane areas [16]. Here we show the effect that this has on LDCV dynamics, providing a unifying model that can describe all apparent vesicle behaviors.

EXPERIMENTAL PROCEDURES

The materials and methods are given in the Supplemental Experimental Procedures.

SUPPLEMENTAL INFORMATION

Supplemental Information includes Supplemental Experimental Procedures, four figures, one table, and three movies and can be found with this article online at http://dx.doi.org/10.1016/j.cub.2016.12.002.

AUTHOR CONTRIBUTIONS

A.R.D., A.M.S., C.R., R.S.W., A.P., D.M.K., K.I.C., L.Y., and R.R.D. acquired and analyzed the data. G.J.L. proposed the mechanism of the model, examined the overlap, and performed the simulations. S.S. and S.P. generated the DKD cells. R.R.D., S.S., A.R.D., and C.R. designed the experiments. R.R.D. and A.R.D. wrote the manuscript.

ACKNOWLEDGMENTS

This work was funded by Medical Research Council grants G0901607 (to R.R.D. and C.R.) and MR/K018639/1 (to C.R.) and Wellcome Trust project grant WT092478MA (to R.R.D and C.R.). We gratefully acknowledge the expertise and infrastructure offered by the Edinburgh Super-Resolution Imaging Consortium (http://www.esric.org). We thank Dr. Sutherland McIver, University of Edinburgh, for his useful conversations about actin biology.

Received: June 8, 2016
Revised: September 5, 2016
Accepted: December 2, 2016
Published: January 12, 2017

REFERENCES

1. Spiller, D.G., Wood, C.D., Rand, D.A., and White, M.R.H. (2010). Measurement of single-cell dynamics. Nature *465*, 736–745.
2. Destainville, N., Schmidt, T.H., and Lang, T. (2016). Where biology meets physics–a converging view on membrane microdomain dynamics. Curr. Top. Membr. *77*, 27–65.
3. Dun, A.R., Rickman, C., and Duncan, R.R. (2010). The t-SNARE complex: a close up. Cell. Mol. Neurobiol. *30*, 1321–1326.
4. Kavanagh, D.M., Smyth, A.M., Martin, K.J., Dun, A., Brown, E.R., Gordon, S., Smillie, K.J., Chamberlain, L.H., Wilson, R.S., Yang, L., et al. (2014). A molecular toggle after exocytosis sequesters the presynaptic syntaxin1a molecules involved in prior vesicle fusion. Nat. Commun. *5*, 5774.
5. Smyth, A.M., Yang, L., Martin, K.J., Hamilton, C., Lu, W., Cousin, M.A., Rickman, C., and Duncan, R.R. (2013). Munc18-1 protein molecules move between membrane molecular depots distinct from vesicle docking sites. J. Biol. Chem. *288*, 5102–5113.
6. Hiersemenzel, K., Brown, E.R., and Duncan, R.R. (2013). Imaging large cohorts of single ion channels and their activity. Front. Endocrinol. (Lausanne) *4*, 114.
7. Won, S., Kim, H.-D., Kim, J.-Y., Lee, B.-C., Chang, S., and Park, C.-S. (2010). Movements of individual BKCa channels in live cell membrane

monitored by site-specific labeling using quantum dots. Biophys. J. *99*, 2853–2862.

8. Bannai, H., Niwa, F., Sherwood, M.W., Shrivastava, A.N., Arizono, M., Miyamoto, A., Sugiura, K., Lévi, S., Triller, A., and Mikoshiba, K. (2015). Bidirectional control of synaptic GABAAR clustering by glutamate and calcium. Cell Rep. *13*, 2768–2780.
9. Degtyar, V.E., Allersma, M.W., Axelrod, D., and Holz, R.W. (2007). Increased motion and travel, rather than stable docking, characterize the last moments before secretory granule fusion. Proc. Natl. Acad. Sci. USA *104*, 15929–15934.
10. Barg, S., Knowles, M.K., Chen, X., Midorikawa, M., and Almers, W. (2010). Syntaxin clusters assemble reversibly at sites of secretory granules in live cells. Proc. Natl. Acad. Sci. USA *107*, 20804–20809.
11. Lang, T., Wacker, I., Steyer, J., Kaether, C., Wunderlich, I., Soldati, T., Gerdes, H.H., and Almers, W. (1997). Ca2+-triggered peptide secretion in single cells imaged with green fluorescent protein and evanescent-wave microscopy. Neuron *18*, 857–863.
12. Rudolf, R., Kögel, T., Kuznetsov, S.A., Salm, T., Schlicker, O., Hellwig, A., Hammer, J.A., 3rd, and Gerdes, H.-H. (2003). Myosin Va facilitates the distribution of secretory granules in the F-actin rich cortex of PC12 cells. J. Cell Sci. *116*, 1339–1348.
13. Purcell, E.K., Liu, L., Thomas, P.V., and Duncan, R.K. (2011). Cholesterol influences voltage-gated calcium channels and BK-type potassium channels in auditory hair cells. PLoS ONE *6*, e26289.
14. Smyth, A.M., Rickman, C., and Duncan, R.R. (2010). Vesicle fusion probability is determined by the specific interactions of munc18. J. Biol. Chem. *285*, 38141–38148.
15. Lang, T., Bruns, D., Wenzel, D., Riedel, D., Holroyd, P., Thiele, C., and Jahn, R. (2001). SNAREs are concentrated in cholesterol-dependent clusters that define docking and fusion sites for exocytosis. EMBO J. *20*, 2202–2213.
16. Khuong, T.M., Habets, R.L.P., Kuenen, S., Witkowska, A., Kasprowicz, J., Swerts, J., Jahn, R., van den Bogaart, G., and Verstreken, P. (2013). Synaptic PI(3,4,5)P3 is required for Syntaxin1A clustering and neurotransmitter release. Neuron *77*, 1097–1108.
17. Rickman, C., Medine, C.N., Dun, A.R., Moulton, D.J., Mandula, O., Halemani, N.D., Rizzoli, S.O., Chamberlain, L.H., and Duncan, R.R. (2010). t-SNARE protein conformations patterned by the lipid microenvironment. J. Biol. Chem. *285*, 13535–13541.
18. Yang, L., Dun, A.R., Martin, K.J., Qiu, Z., Dunn, A., Lord, G.J., Lu, W., Duncan, R.R., and Rickman, C. (2012). Secretory vesicles are preferentially targeted to areas of low molecular SNARE density. PLoS ONE *7*, e49514.
19. Medine, C.N., Rickman, C., Chamberlain, L.H., and Duncan, R.R. (2007). Munc18-1 prevents the formation of ectopic SNARE complexes in living cells. J. Cell Sci. *120*, 4407–4415.
20. Betzig, E., Patterson, G.H., Sougrat, R., Lindwasser, O.W., Olenych, S., Bonifacino, J.S., Davidson, M.W., Lippincott-Schwartz, J., and Hess, H.F. (2006). Imaging intracellular fluorescent proteins at nanometer resolution. Science *313*, 1642–1645.
21. Hess, S.T., Girirajan, T.P.K., and Mason, M.D. (2006). Ultra-high resolution imaging by fluorescence photoactivation localization microscopy. Biophys. J. *91*, 4258–4272.
22. van de Linde, S., Löschberger, A., Klein, T., Heidbreder, M., Wolter, S., Heilemann, M., and Sauer, M. (2011). Direct stochastic optical reconstruction microscopy with standard fluorescent probes. Nat. Protoc. *6*, 991–1009.
23. Riedl, J., Crevenna, A.H., Kessenbrock, K., Yu, J.H., Neukirchen, D., Bista, M., Bradke, F., Jenne, D., Holak, T.A., Werb, Z., et al. (2008). Lifeact: a versatile marker to visualize F-actin. Nat. Methods *5*, 605–607.
24. Meunier, F.A., and Gutiérrez, L.M. (2016). Captivating new roles of F-Actin cortex in exocytosis and bulk endocytosis in neurosecretory cells. Trends Neurosci. *39*, 605–613.
25. Nofal, S., Becherer, U., Hof, D., Matti, U., and Rettig, J. (2007). Primed vesicles can be distinguished from docked vesicles by analyzing their mobility. J. Neurosci. *27*, 1386–1395.
26. Voets, T., Toonen, R.F., Brian, E.C., de Wit, H., Moser, T., Rettig, J., Südhof, T.C., Neher, E., and Verhage, M. (2001). Munc18-1 promotes large dense-core vesicle docking. Neuron *31*, 581–591.
27. Toonen, R.F.G., de Vries, K.J., Zalm, R., Südhof, T.C., and Verhage, M. (2005). Munc18-1 stabilizes syntaxin 1, but is not essential for syntaxin 1 targeting and SNARE complex formation. J. Neurochem. *93*, 1393–1400.
28. Han, L., Jiang, T., Han, G.A., Malintan, N.T., Xie, L., Wang, L., Tse, F.W., Gaisano, H.Y., Collins, B.M., Meunier, F.A., and Sugita, S. (2009). Rescue of Munc18-1 and -2 double knockdown reveals the essential functions of interaction between Munc18 and closed syntaxin in PC12 cells. Mol. Biol. Cell *20*, 4962–4975.
29. de Wit, H., Walter, A.M., Milosevic, I., Gulyás-Kovács, A., Riedel, D., Sørensen, J.B., and Verhage, M. (2009). Synaptotagmin-1 docks secretory vesicles to syntaxin-1/SNAP-25 acceptor complexes. Cell *138*, 935–946.
30. de Wit, H., Cornelisse, L.N., Toonen, R.F., and Verhage, M. (2006). Docking of secretory vesicles is syntaxin dependent. PLoS ONE *1*, e126.
31. Manley, S., Gillette, J.M., Patterson, G.H., Shroff, H., Hess, H.F., Betzig, E., and Lippincott-Schwartz, J. (2008). High-density mapping of single-molecule trajectories with photoactivated localization microscopy. Nat. Methods *5*, 155–157.
32. Wilson, R.S., Yang, L., Dun, A., Smyth, A.M., Duncan, R.R., Rickman, C., and Lu, W. (2016). Automated single particle detection and tracking for large microscopy datasets. R. Soc. Open Sci. *3*, 160225.
33. Bar-On, D., Gutman, M., Mezer, A., Ashery, U., Lang, T., and Nachliel, E. (2009). Evaluation of the heterogeneous reactivity of the syntaxin molecules on the inner leaflet of the plasma membrane. J. Neurosci. *29*, 12292–12301.
34. Rickman, C., Medine, C.N., Bergmann, A., and Duncan, R.R. (2007). Functionally and spatially distinct modes of munc18-syntaxin 1 interaction. J. Biol. Chem. *282*, 12097–12103.
35. Kasula, R., Chai, Y.J., Bademosi, A.T., Harper, C.B., Gormal, R.S., Morrow, I.C., Hosy, E., Collins, B.M., Choquet, D., Papadopulos, A., and Meunier, F.A. (2016). The Munc18-1 domain 3a hinge-loop controls syntaxin-1A nanodomain assembly and engagement with the SNARE complex during secretory vesicle priming. J. Cell Biol. *214*, 847–858.
36. Honigmann, A., van den Bogaart, G., Iraheta, E., Risselada, H.J., Milovanovic, D., Mueller, V., Müllar, S., Diederichsen, U., Fasshauer, D., Grubmüller, H., et al. (2013). Phosphatidylinositol 4,5-bisphosphate clusters act as molecular beacons for vesicle recruitment. Nat. Struct. Mol. Biol. *20*, 679–686.
37. Saka, S.K., Honigmann, A., Eggeling, C., Hell, S.W., Lang, T., and Rizzoli, S.O. (2014). Multi-protein assemblies underlie the mesoscale organization of the plasma membrane. Nat. Commun. *5*, 4509.
38. Knowles, M.K., Barg, S., Wan, L., Midorikawa, M., Chen, X., and Almers, W. (2010). Single secretory granules of live cells recruit syntaxin-1 and synaptosomal associated protein 25 (SNAP-25) in large copy numbers. Proc. Natl. Acad. Sci. USA *107*, 20810–20815.

Current Biology, Volume *27*

Supplemental Information

Navigation through the Plasma Membrane Molecular Landscape Shapes Random Organelle Movement

Alison R. Dun, Gabriel J. Lord, Rhodri S. Wilson, Deirdre M. Kavanagh, Katarzyna I. Cialowicz, Shuzo Sugita, Seungmee Park, Lei Yang, Annya M. Smyth, Andreas Papadopulos, Colin Rickman, and Rory R. Duncan

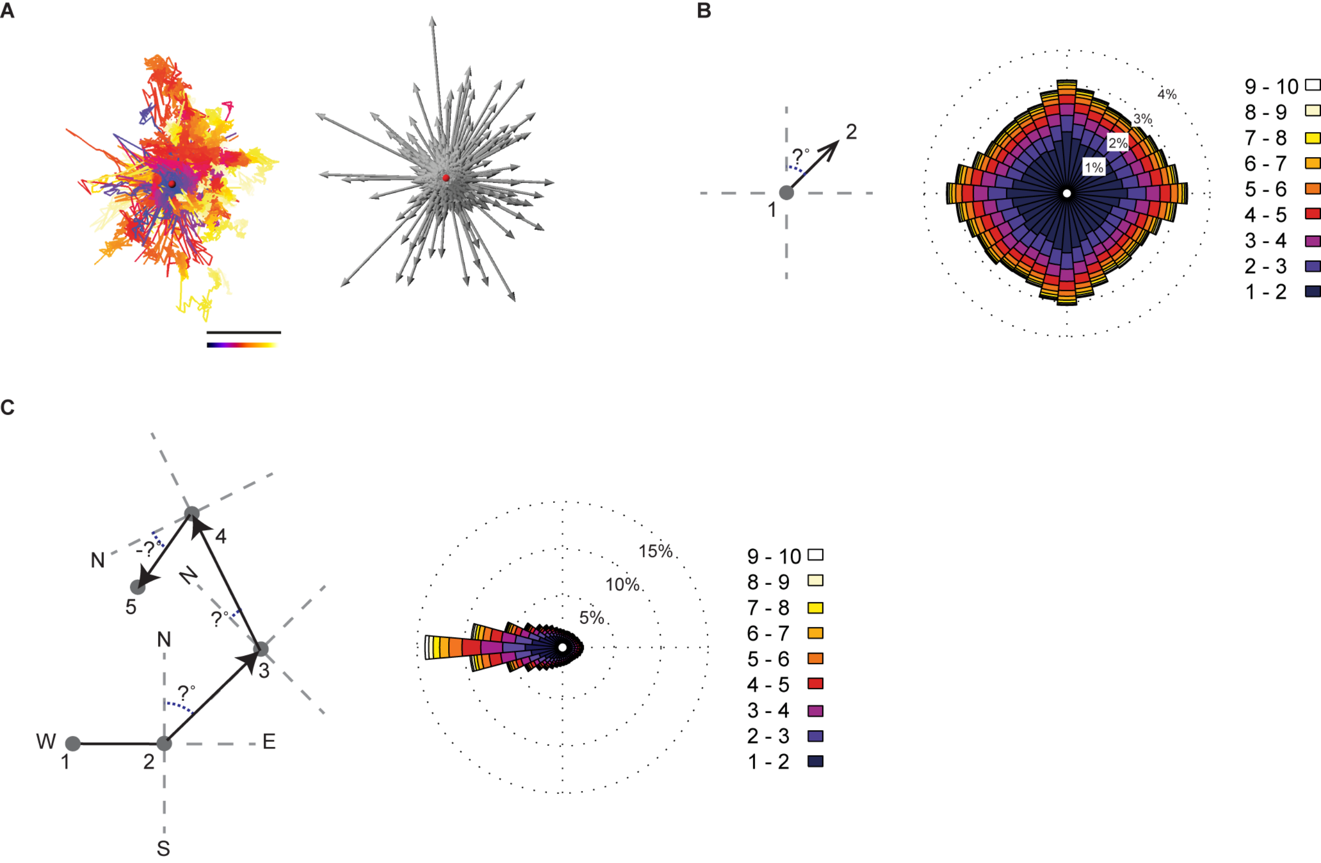

A
B
?°
2
1
4%
3%
2%
1%
9 - 10
8 - 9
7 - 8
6 - 7
5 - 6
4 - 5
3 - 4
2 - 3
1 - 2
C
N
-?°
4
5
N
?°
N
?°
3
W
1
2
E
S
15%
10%
5%
9 - 10
8 - 9
7 - 8
6 - 7
5 - 6
4 - 5
3 - 4
2 - 3
1 - 2

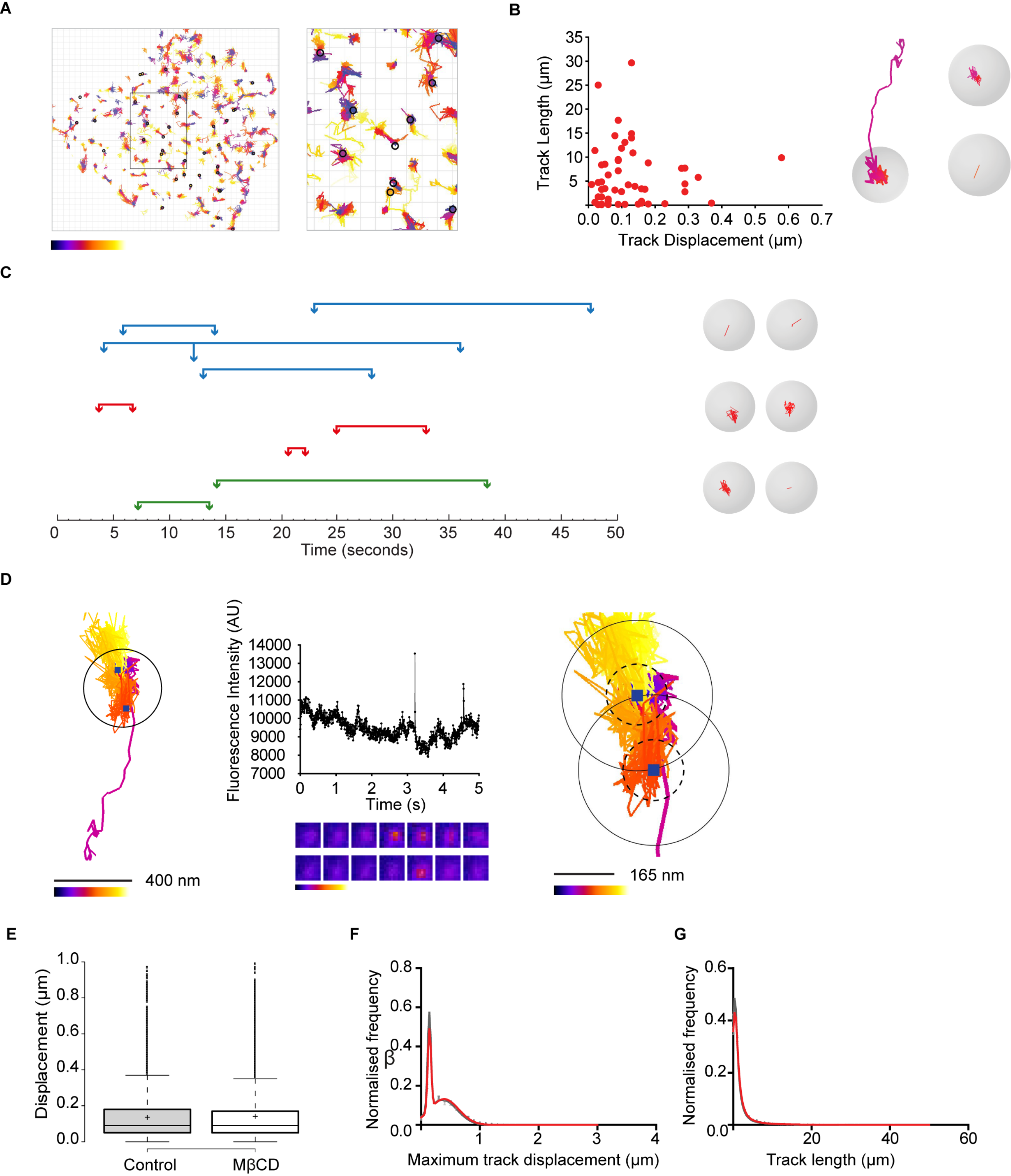
A
B
Track Length (μm)
35
30
25
20
15
10
5
0.0
0.1
0.2
0.3
0.4
0.5
0.6
0.7
Track Displacement (μm)
C
0
5
10
15
20
25
30
35
40
45
50
Time (seconds)
D
400 nm
Fluorescence Intensity (AU)
14000
13000
12000
11000
10000
9000
8000
7000
0
1
2
3
4
5
Time (s)
165 nm
E
Displacement (μm)
1.0
0.8
0.6
0.4
0.2
0.0
Control
MβCD
F
Normalised frequency
0.8
0.6
0.4
0.2
0.0
β
1
2
3
4
Maximum track displacement (μm)
G
Normalised frequency
0.6
0.4
0.2
0.0
20
40
60
Track length (μm)

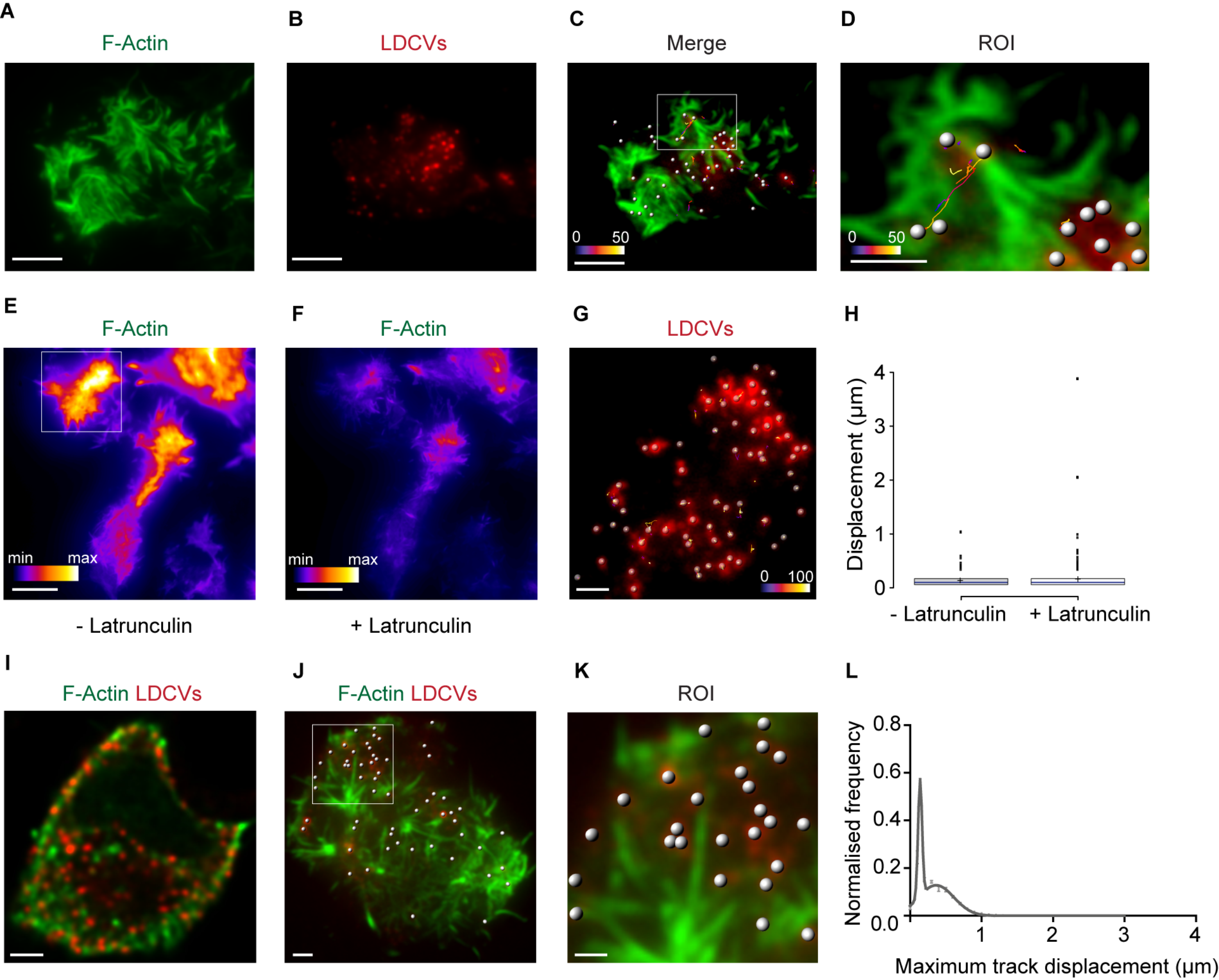
A
F-Actin
B
LDCVs
C
Merge
D
ROI
0
50
E
F-Actin
min
max
F
F-Actin
G
LDCVs
0
100
H
Displacement (μm)
- Latrunculin
+ Latrunculin
I
F-Actin LDCVs
J
F-Actin LDCVs
K
ROI
L
Normalised frequency
Maximum track displacement (μm)

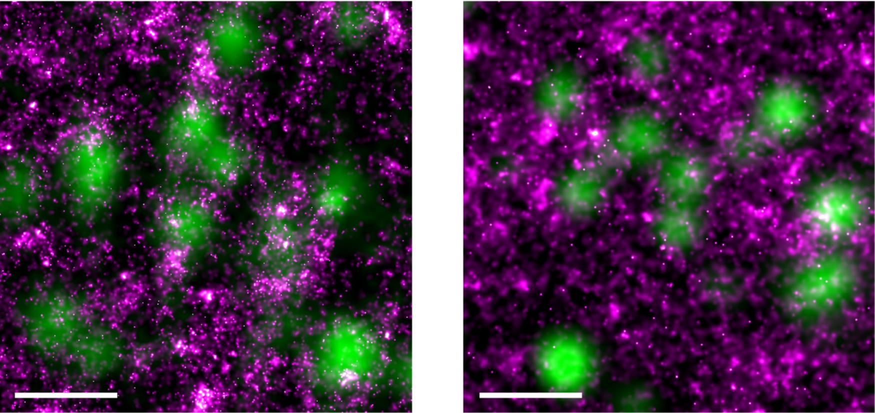
Control Cell
Munc18-1/2 DKD Cell

Supplemental Figure Legends

Figure S1 Vesicle mobility can be described by a Brownian model (related to Figure 1). A. Vesicle tracks (829) from a representative PC12 cell over 100 seconds, translated to start at the same xy coordinate. Coloured vesicle tracks (left) and track displacement displayed as arrows (right). Scale bar 1 µm. B. the angle and speed of the first movement taken by a vesicle is extracted (left; vesicle; grey circle, movement; black arrow, angle; blue dashed line) and displayed as a histogram (right) where colour represents speed (µm s^{-1}) and length of the bin represents frequency (as described in figure 4). Histogram shows 16,944 tracks from 5 representative PC12 cells. C. The angle and speed of the subsequent movements a vesicle makes in relation to its previous is extracted (left; as in b with subsequent steps numbered and compass points used for perspective) and displayed as a histogram (as in b). Colours indicate binned speeds, units are microns per second.

Figure S2 Vesicles fuse at frequently visited sites on the plasma membrane (related to Figure 2). Where appropriate, cells were stimulated to secrete with 300 µM ATP, added to the bath after 500 frames imaging at 20 Hz, with imaging continued for 1000 further frames. Secretion was visualised using TIRFM, by observing 'flashes' as luminal, acidic NPY-EGFP is exposed to the higher, extracellular pH. A. tracked vesicles (coloured tracks) at the membrane of a representative PC12 cell are shown, black circles show vesicle fusion sites within a 45 second recording, grid shows scale 400 nm. Black box indicates zoomed region (right panel). B. Track length against track displacement for vesicles that fused shows range of behaviour, example tracks shown (grey spheres show vesicle). C. arrow heads represent points of fusion along the scale with cell stimulation at 0 seconds, line shows refractory period until subsequent fusion in same 400 nm^2 region. Example tracks (red) within vesicles (grey sphere) show these vesicle pairs can immediately fuse (blue, top right), show caged behaviour (red, middle right) or a combination (green, lower right). D. (left panel) two vesicle fusion sites within 400 nm radius (solid circle), (middle panel) profile of fluorescence intensity over time (colour represents intensity). (right panel) fusion sites using radius of 82.5 nm (dashed line) are separate. All vesicle tracks are colour-coded to show time from 0 – 45 seconds (excluding C). E. Quantification of the effect of MβCD on PC12 cells. Box plots show displacement for all vesicles tracked inside wt PC12 and MβCD-treated PC12 cells (5 control cells and 6 MβCD-treated PC12 cells). Line shows median displacement, cross hair shows mean displacement and outliers are presented. F - G. maximum track displacement and track length shown from PC12 cells (grey) and MβCD-treated PC12 cells (red). Data normalised to number of tracks per cell (mean +/- SEM) n.s between control and treated in either parameter using a Mann-Whitney test.

Figure S3 Actin and vesicle imaging at the plasma membrane (related to Figure 3). A. F-actin and B. LDCVs at the plasma membrane of wtPC12 cells under TIRF illumination. C. a merge of both components with vesicles tracked at 20 Hz. Scale bar 5 µm. D. zoom of region of interest showing vesicles moving perpendicular to actin fibres, scale bar 2 µm. Tracks coloured as per colour bar from 0 to 50 frames. E. F-actin in wtPC12 cells under TIRF illumination before Latrunculin treatment and F. after treatment. Scale bar 10 µm, false colour showing intensity (colour bar min to max). G. LDCVs at the plasma membrane of the cell highlighted in e. tracked over 100 frames at 14 Hz. Scale bar 2 µm, Tracks coloured as per colour bar from 0 to 100 frames. H. Vesicle displacement from vesicles tracked across field of view in E. Median (blue line) 0.1 µm for both before and after treatment, mean (cross) 0.14 µm and 0.17 µm for before and after treatment. Outliers shown. I. Equatorial section of a PC12 cell showing F-actin and LDCVs imaged under CLSM and deconvolved. Scale bar 2 µm. J. a merge of both components under TIRF illumination with vesicles identified (grey spheres). Scale bar 2 µm. K. zoom into region of interest showing vesicles in gaps of actin >1 µm. Scale bar 1 µm. L. the distribution of the maximum displacement length of vesicle tracks from 16,944 tracks in 5 PC12 cells over 2 minute recordings, error bars show SEM.

Figure S4 Syntaxin and vesicle organisation at the plasma membrane (related to Figure 2 and Figure 3) A dSTORM image map showing the nano-scale positions of immuno-detected (magenta) syntaxin1 molecules in a region of plasma membrane in wild-type (*left* panel) and munc18-1/2 DKD cells (*right*), with the positions of LDCVs shown in green. Scale bars: 1 µm.

Table S1 *in silico* model parameters are predictive

6 different measures of LDCV behavior are presented, with the difference between *in silico* and real control *versus* mutant cells (related to Figure 3).

	Max Dist from EXY	E Dist from XY0	Max Dist XY0	MSD from EXY	MSD from XY0
	0.80 ± 0.04	0.69 ± 0.003	1.09 ± 0.03	0.08± 0.008	0.60 ± 0.01
Control – Mutant distance	**0.80**	**0.69**	**1.09**	**0.08**	**0.61**
	0.20 ± 0.02	0.12 ± 0.02	0.25 ±	0.01 ± 0.007	0.06 ± 0.06

			0.03		
Control – Mutant distance	**0.19**	**0.12**	**0.25**	**0.01**	**0.06**

Explanation of terms:

Max dist from EXY = maximum distance from the mean position
E dist from XY0 = average distance from the starting position
Max dist from XY0 = maximum distance from the starting position
MSD from EXY = mean squared distance from mean position.
MSD from XY0 = mean squared distance from starting position

All units are microns.

In all cases, the sign of the difference between control versus mutant values is the same (i.e. positive in these experiments), indicating the predictive nature of the in silico model. The positive sign here predicted that the vesicles in the mutant cells would become more mobile, as observed.

Supplemental Experimental Procedures

Cell culture, plasmids and transfections

Munc18-1 siRNA PC-12 cells (DKD and KD43) were a kind gift of Shuzo Sugita[S1] and grown in DMEM medium supplemented with 5% (v/v) horse serum, 5% (v/v) fetal bovine serum, 1% (v/v) Penicillin/Strep (Invitrogen), 400 µg/ml G418, 2. 5 µg/ml puromycin and maintained at 37 °C in 7.5% (v/v) CO_2, 92.5% (v/v) air. LDCVs were labelled using NPY-EGFP as previously described[S2]. Transfections were performed using Lipofectamine 2000 (Invitrogen). Where appropriate, cells were stimulated to secrete with 300 µM ATP, added to the bath after 500 frames imaging at 20 Hz, with imaging continued for 1000 further frames. Secretion was visualised using TIRFM as we have described before[S3], by observing 'flashes' as lumenal, acidic NPY-EGFP is exposed to the higher, extracellular pH. Lifeact-EGFP and NPY-mCherry were used for F-actin experiments, Latrunculin-A at 15 µM was added to the cells whilst imaging to disrupt actin.

Single molecule imaging

Cells were transfected with either Munc18-1, SNAP-25 or syntaxin1a, fused to PAmCherry and fixed with 4% (w/v) paraformaldehyde, 0.1% (w/v) glutaraldehyde for 90 minutes. PALM acquisition involved cycles of brief activation at 405 nm followed by rapid imaging in TIRF mode at 561 nm. All PALM and dSTORM experiments were performed using an Olympus IX-81 microscope equipped with Olympus Cell^R acquisition software, an ImageEM EM-CCD 512x512 camera (Hamamatsu UK) and an Olympus 150X UAPO 1.45NA oil lens with a resulting pixel size of 106 nm. For dSTORM, endogenous proteins were immunolabelled, after 90-minute fixation in 4% (w/v) buffered paraformaldehyde with primary antibodies (syntaxin1a (HPC-1)). Immunodetected syntaxin1a molecules were subsequently labelled with Alexa-647-conjugated anti-IgG (Invitrogen). Alexa-647 was found to have the greatest propensity to enter a dark state using 640 nm illumination in an oxygen-depleting buffer (50 mM MEA in PBS buffered at pH 7.4).

For sptPALM, the image acquisition regime was similar to above, except live cells were used. Imaging was performed at 37°C in 7.5% (v/v) CO_2 with brief activation pulses at 405 nm followed by trains of 100 frames with 561 nm excitation, with a 30 ms exposure time.

Single molecule detection and tracking was performed essentially as we previously described[S4].

The performance metrics were calculated to have equivalency with the values determined in [S5] from synthetic image data used in [S6].
The signal to noise ratio of the datasets were calculated using an intensity based method; $SNR = (I_0 - I_b)/\sqrt{I_0}$, where I_0 and I_b denote the peak object intensity and the mean background intensity respectively as in [S6].

Confocal imaging

Confocal laser-scanning microscope images were acquired at Nyquist sampling rates using a Leica SP5-SMD inverted system, equipped with a white-light super-continuum laser excitation source using a 63X 1.2NA water immersion lens. EGFP and mCherry were excited at 490 nm and 560 nm, respectively, and emission collected between 500-550 nm and 590 – 670 nm. Image data were subsequently deconvolved using Huygens Pro software (SVI, NL), using a theoretical point-spread-function. Image data were visualised using Imaris (Andor, UK).

Fluorescence Correlation Spectroscopy

Prior to each FCS experiment the values for *Kappa* (the ratio of the axial and waist excitation spot dimensions) and V_{eff} were determined using 10 nM Atto488 and Atto561 (Atto-Tec GmbH, Germany) standards in water at either 25°C or 37°C (Supplemental Figure 3). Atto488 has a well-established diffusion rate of 400 $\mu m^2 s^{-1}$ (Picoquant). The resulting Veff and K were verified using 10 nM purified EGFP and mCherry proteins (25kDa) and 10 nM fused EGFP-mCherry protein (50 kDa) in 150 mM NaCl, 20 mM Tris pH7.4, 1 mM DTT and 0.1% (v/v) Tween 20. The resulting diffusion coefficients of the fluorescent proteins (EGFP and mCherry of 124 ± 0.17 and 106 ± 0.12 $\mu m^2 s^{-1}$ at 37°C respectively n=10) are consistent with Stokes-Einstein (where diffusion is inversely proportional to the hydrodynamic radius of the molecule and the viscosity of the surrounding media) estimated diffusion coefficients of 25 kDa proteins under these conditions. Calibration recordings of 30 seconds were made for each standard. These calibrations determined that the effective volume of the FCS spot was 0.29 ± 0.04 μm^3 at 37°C; all *in cellulo* FCS measurements were made at this temperature.

FCS Analyses
Autocorrelation traces were generated from the photon counting histograms for each 5 to 30 s measurement using SymPhoTime v5.4.4 software (Picoquant GmbH, Germany). *In vitro* calibration traces were fitted using the Triplet model (3D free diffusion model with triplet state) with informed diffusion values to yield Veff and Kappa values. Neuronal autocorrelation traces were fitted using a Triplet Extended model (2-D anomalous diffusion model with triplet state), this model is designed for fluorescent molecules moving within a plane e.g. proteins in a membrane. Diffusion within cells is expected to be anomalous therefore the anomaly parameter was not fixed to one. The anomaly parameter (α) measures the departure from free Brownian diffusion (α =1) to either super-diffusion (α >1) or subdiffusion (α <1) for a diffusing species. Autocorrelation curves with (α >1) display the sharpest decay, whereas the those with α <1 decrease quite slowly. The goodness-of-fit was examined by inspection of the residuals, which should be randomly distributed around zero.

Mathematical modelling
We consider the movement of vesicles in a potential field V formed by the presence of molecules such as SNARE/munc-18. We use for this a classic Langevin type equation that models a large particle immersed in a fluid of smaller ones given by

$$dq = p\, dt$$
$$dp = (B\, p - \alpha\, \mathrm{grad}(V(q)))\, dt + \sigma dW$$

We use a classic Langevin equation that models a large particle (assumed here to have unit mass) in a fluid;
B=-0.82 [1/s], alpha=5 [mu Kg/s^2], sigma=0.6 [Kg mu / s sqrt(s)]
B=-0.82 [1/s], alpha=1.25 [mu Kg/s^2], sigma=0.6 [Kg mu / s sqrt(s)]
mu=micro meters.
q=(q_1, q_2)^T is the displacement and where alpha is the weight of the forcing. The term Bp represents a friction term (since B is taken to be negative) and σdW models the random collisions with smaller molecules in the cell. W is a standard Brownian motion.

We construct the potential field V from real biological data on the positions of molecules in a particular cell imaged using PALM. At each molecule position we have the peak of a two dimensional Gaussian function centred on this position. Other forms of potential were also investigated but the Gaussian was kept for simplicity. The field is scaled so that max(V(q)) =1 and min(V(q))=0 and the radius of the molecule (taken to be 5e-3 microns) was matched to a potential of approximately > 0.9. This yields a potential field such as shown, for example, in Figure 2. We do not impose boundary conditions on the computational domain, instead, once a vesicle leaves the computational domain its track is no longer considered. Initial data for the vesicle positions q(0) were determined from corresponding PALM data and all synthetic vesicles were started with an initial velocity of zero.

Numerical implementation.
Given the potential field V it remains to find appropriate parameters for the friction term B, the strength of the stochastic forcing σ and weight of the potential α. For the control data set we take: B=-0.82, α =5 and σ = 0.6 whereas for the mutant data set we take B=-0.82, α =1.25 and σ = 0.6. That is we assume the same friction, same noise term and simply reduce the strength of potential field (and hence influence of the molecules) by 1/4. Initially a least-squares fit to the observed mean(log(average area)) was examined for the control data this leads to different parameter values (B=-1.18, α = 9.59, σ = 0.6), however the variance around the mean was not realistic. Here average area refers to the maximum distance travelled by the vesicle from its average position within its track, scaled by tracklength. The parameter values actually used were then determined by hand for the control. For the mutant the single parameter α was used to obtain the results. This gives weight to the idea that a change in the potential field of surrounding molecules is a key difference between the mutant and control.

In implementation a spline interpolant was used for second order finite difference approximations of the derivatives of potential V(q) as this proved computationally more efficient. Statistics on track length and distance are determined

using the same time scales T0 between frames for the experimental data (50x1e-3 s for the control and 70x1e-3 for the mutant) and the final time solved for was the same as in the experiments (corresponding to 500 and 358 frames respectively). However, the tracks of the modelled vesicles are resolved at smaller time scale than is feasible in the experiments and in both cases we took T0/40 as our numerical step size. Standard (explicit and semi-implicit) Euler-Maruyama type schemes were used to integrate the Langevin equations. We examined 100 vesicles arriving at six different initial positions in the computational domain leading to 600 vesicles for each realization of the cell. The simulation was then performed 4 times to compute statistics.

Overlap. The potential field V for the molecules may be interpreted as a probability field for having a molecule at a particular position – thus if $V(q)>0.9$ it is highly likely that a molecule would be found. We can also construct a probability field for the positions of the vesicles with an in-plane radius of 82.5 microns. We can then consider the probability of an overlap between the molecules and the vesicles by examining the area covered by the molecules and vesicles and by subtracting.
For cellular data, we examined the probability the vesicles and molecules do not overlap. This was done for squares inside the cell with different side lengths (e.g. 1,2,3,4,5,6 microns). In total we take 7 sizes on two different parts of each of the cells to get an average chance of not overlapping (as in Figure 2).

Mean-squared displacement (MSD) is typically used to identify if displacement from a fixed (typically initial) position grows linearly in time, indicating that a particle is undergoing Brownian motion, looking at a distance (RMSD) travelled then simply changes the scaling. We have introduced in this article novel ideas for examining the time series data of particle movement. In Fig. 3 we look at "Average Area" which captures how far the vesicle moves from its mean position i.e. does it travel a long distance or a short distance from where you expect it to be.

Statistical methods
Statistical analyses were performed using GraphPad Prism. Data sets were first tested for normality using the Shapiro-Wilks test. Data that fitted a normal distribution was tested for statistical significance by two-tailed unpaired Students t-test. Failing Normality, the data were analysed using the Mann-Whitney test. All histogram data are presented as standard deviation of the mean (±S.D.) and boxplot data as min and max data, for completeness. Boxplots were created using BoxPlotR tools [S8]. We estimated the statistical power of our conclusions where appropriate and for vesicle and single-molecule dynamic comparisons, found all datasets to provide a power of >99% at the replicate number we employed using a post-hoc power calculation[S9]. For the data in Figure 3d, the data were tested for Normality using the Shapiro-Wilks test, then a 2-way ANOVA with the Kuskal-Wallace test.

Supplemental references

S1. Arunachalam, L., Han, L., Tassew, N. G., He, Y., Wang, L., Xie, L., Fujita, Y., Kwan, E., Davletov, B., Monnier, P. P., et al. (2008). Munc18-1 Is Critical for Plasma Membrane Localization of Syntaxin1 but Not of SNAP-25 in PC12 Cells. Mol Biol Cell *19*, 722–734.

S2. Rickman, C., Medine, C. N., Bergmann, A., and Duncan, R. R. (2007). Functionally and spatially distinct modes of munc18-syntaxin 1 interaction. J. Biol. Chem. *282*, 12097–103.

S3. Smyth, A. M., Rickman, C., and Duncan, R. R. (2010). Vesicle fusion probability is determined by the specific interactions of munc18. J. Biol. Chem. *285*, 38141–8.

S4. Yang, L., Dun, A. R., Martin, K. J., Qiu, Z., Dunn, A., Lord, G. J., Lu, W., Duncan, R. R., and Rickman, C. (2012). Secretory Vesicles Are Preferentially Targeted to Areas of Low Molecular SNARE Density. PLoS One *7*, e49514.

S5. Wilson, R. S., Yang, L., Dun, A., Smyth, A. M., Duncan, R. R., Rickman, C., and Lu, W. (2016). Automated single particle detection and tracking for large microscopy datasets. R. Soc. open Sci. *3*, 160225.

S6. Chenouard, N., Smal, I., de Chaumont, F., Maška, M., Sbalzarini, I. F., Gong, Y., Cardinale, J., Carthel, C., Coraluppi, S., Winter, M., et al. (2014). Objective comparison of particle tracking methods. Nat. Methods *11*, 281–9.

S7. Wilhelm, B. G., Mandad, S., Truckenbrodt, S., Kröhnert, K., Schäfer, C., Rammner, B., Koo, S. J., Claßen, G. A., Krauss, M., Haucke, V., et al. (2014). Composition of isolated synaptic boutons reveals the amounts of vesicle trafficking proteins. Science *344*, 1023–8.

S8. Spitzer, M., Wildenhain, J., Rappsilber, J., and Tyers, M. (2014). BoxPlotR: a web tool for generation of box plots. Nat. Methods *11*, 121–2.

S9. Levine, M., and Ensom, M. H. (2001). Post hoc power analysis: an idea whose time has passed? Pharmacotherapy *21*, 405–9.
